# A Permanent Automated Real-Time Passive Acoustic Monitoring System for Bottlenose Dolphin Conservation in the Mediterranean Sea

**DOI:** 10.1371/journal.pone.0145362

**Published:** 2016-01-20

**Authors:** Marco Brunoldi, Giorgio Bozzini, Alessandra Casale, Pietro Corvisiero, Daniele Grosso, Nicodemo Magnoli, Jessica Alessi, Carlo Nike Bianchi, Alberta Mandich, Carla Morri, Paolo Povero, Maurizio Wurtz, Christian Melchiorre, Gianni Viano, Valentina Cappanera, Giorgio Fanciulli, Massimiliano Bei, Nicola Stasi, Mauro Taiuti

**Affiliations:** 1 Department of Physics, University of Genova, Genova, Italy; 2 Department of the Earth, Environment and Life Science, University of Genova, Genova, Italy; 3 SOFTECO Sismat S.r.l, Genova, Italy; 4 Area Marina Protetta di Portofino, Ministry for the Environment and for the Protection of Territory and Sea, Santa Margherita Ligure, Genova, Italy; 5 Direzione Marittima di Genova, Ministry for the Infrastructures and Transport, Genova, Italy; University of Missouri, UNITED STATES

## Abstract

Within the framework of the EU Life+ project named LIFE09 NAT/IT/000190 ARION, a permanent automated real-time passive acoustic monitoring system for the improvement of the conservation status of the transient and resident population of bottlenose dolphin (*Tursiops truncatus*) has been implemented and installed in the Portofino Marine Protected Area (MPA), Ligurian Sea. The system is able to detect the simultaneous presence of dolphins and boats in the area and to give their position in real time. This information is used to prevent collisions by diffusing warning messages to all the categories involved (tourists, professional fishermen and so on). The system consists of two gps-synchronized acoustic units, based on a particular type of marine buoy (elastic beacon), deployed about 1 km off the Portofino headland. Each one is equipped with a four-hydrophone array and an onboard acquisition system which can record the typical social communication whistles emitted by the dolphins and the sound emitted by boat engines. Signals are pre-filtered, digitized and then broadcast to the ground station via wi-fi. The raw data are elaborated to get the direction of the acoustic target to each unit, and hence the position of dolphins and boats in real time by triangulation.

## Introduction

Amongst Mediterranean cetaceans, bottlenose dolphin (*Tursiops truncatus*) is highly exposed to threats due to human activities and resource exploitation. As being a coastal species it suffers the impacts of the human activity more than other species. The main threats are due to coastal urbanization, ports construction, boat traffic, shipping, pollution by industrial and agriculture activities, over fishing and overexploitation. This factor made it protected under the Annex II, Habitat directive [1], in which it is included. This directive requires member states to select, designate and protect sites that support specific natural habitats or species of plants or animals as Special Areas of Conservation (SACs) that aim to create a network of protected areas across the European Union (EU) known as Natura 2000. In the same report, the areas with high level of tourism activity and fishing are pointed out as the areas where effective conservation actions are needed. Most of the threats (collisions, entanglement in fishnets and disturbance of feeding or breeding activities) are related to the lack of knowledge of the presence and movements of individuals. The Mediterranean population is also present in the IUCN Red List where it is recognized as Vulnerable, according to criteria A2, c, d, e. Criteria A indicates a reduction in population size based on an observed, estimated, inferred or suspected population size reduction of greater than 30% over the last 10 years or three generations, whichever is the longer, where the reduction or its causes may not have ceased or may not be understood or may not be reversible, based on (and specifying) any of (a) to (e) under A1 (sub-criteria A2). The trend of Mediterranean bottlenose dolphins population is decreasing under the following sub-criteria:

(c) a decline in area of occupancy, extent of occurrence and/or quality of habitat;
(d) actual or potential levels of exploitation;
(e) the effects of introduced taxa, hybridization, pathogens, pollutants, competitors or parasites.

This study takes into account the following threat: “boat traffic, including leisure craft that can lead to collisions and noise pollution” [2]. Impacts of boat activity on marine mammals are of particular concern in coastal areas because of the large number of boats, their widespread use, high noise level, speed, and mobility [3]. Boats pose both direct and indirect threats to dolphins. Boats can cause dolphins to change movement patterns, alter behaviour, or can even collide with dolphins [4]. Powerboats emit high amplitude that is, continuous underwater noise that could disrupt echolocation, mask communication, or cause temporary or permanent physical damage to a dolphins ears [5]. Indirect effects of boat traffic include influencing prey movement, degrading habitat quality, or causing avoidance of critical feeding or breeding areas [3]. There is growing evidence that prolonged direct (or physical) disturbance and noise caused by boat traffic can affect the behavior and habitat use of bottlenose dolphins [6, 7]. Various alterations in behavior have been shown to be related to boat disturbance in groups of bottlenose dolphins [8–15]. [8] reported that the dominant behavioural responses of cetaceans to boat traffic were an increase in swim velocity, spatial avoidance, and change in diving patterns. Permanent or temporary avoidance of one area by bottlenose dolphins as a consequence of seasonal increase in boat traffic was reported in several places around the world [16–19], also in the Mediterranean area (International Whaling Commission, 2007). The core of the ARION project is a permanent automated real-time passive acoustic monitoring (PAM) system aimed at the detection and tracking of dolphins and boats transiting the monitoring area. It is located about 1 km off the south coast of the Portofino promontory (Ligurian sea) and its monitoring range embraces part of the Portofino Marine Protected Area (MPA). Within the field of cetacean research, a passive acoustic monitoring (PAM) system can be defined as a set of acoustic and electronic devices aimed at detecting and tracking marine mammals by listening to their vocalizations. The word “passive” in the acronym is related to the fundamental component of a PAM system, the hydrophone, which is a totally passive pressure-to-voltage transducer. Since no artificial underwater sound is produced by the system, the monitoring of the cetaceans does not interfere with their daily activities (preying, orientation, social communication).

The Portofino MPA, established in 1999, covers a marine area of 346 ha and includes threes zones with decreasing protection level: zone A, strict reserve (no take no entry); zone B, general reserve; zone C, partial reserve [20]. A synopsis of the marine ecosystems occurring in the area before and after the enforcement of the MPA can be found in [21] and [22]. Within its detection area the PAM system is capable to track dolphins and boats. Portofino MPA represents a core area for bottlenose dolphins [23], which regularly visit the area [24], and boat noise and physical presence are known to represent a major disturbance to them [25]. The system aims at identifying the threats and preventing collisions and other risks by diffusing presence warning messages in real time to all the categories involved (tourists, professional and recreational fishermen, MPA management). A protocol of conduct for reducing risks for the species has been developed by the local Coast Guard branch in collaboration with involved stakeholders. The ship and boat owners present in the area are invited to follow the protocol of conduct. The Coast Guard is responsible for overseeing its application. This approach should ensure the species protection improvement, the sustainable coexistence of dolphins and anthropic activities and will promote responsible usage of the sea. The system should also provide the national authorities with the information required to update rules and “Special Area of Conservation” boundaries [26] necessary to improve preservation measures of the species.

## Materials and Methods

All of the activities carried out within the project area have been approved by the competent authorities. This study involves a particular protected species (bottlenose dolphin) but it does not make use of any experimental method potentially harmful for the animal. The acoustic monitoring system, object of this study, is based on the recording by underwater hydrophones of the acoustic signals emitted by dolphins. The system is totally passive therefore there is no artificial sound that can potentially disturb the dolphin’s communication or orientation signals. Conversely the whole system aims to the improvement of the conservation status of the bottlenose dolphin as requested by the European Commission to the beneficiaries partners of the LIFE+ project. For these reasons, no approval was required by any animal ethics committee for this study.

### Acoustic system overview

**Fig 1:**
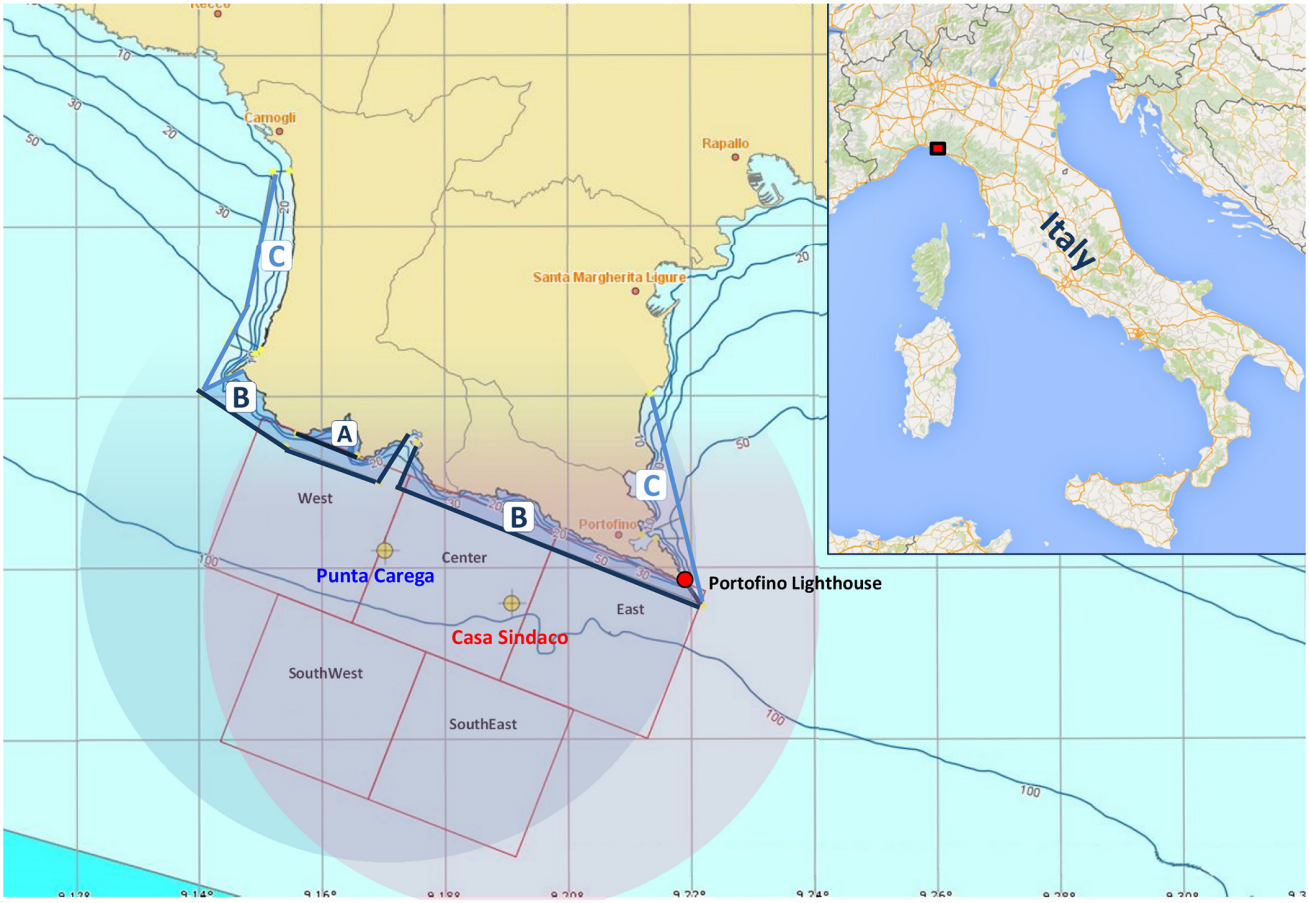
Map of the study area. The boundaries of the Portofino MPA and the position of the three different zones with restricted acces are reported on the map. The round areas centered on the two acoustic units represent the monitoring area of the PAM system.

The system consists of two autonomous acoustic units deployed next to the boundaries of the Portofino MPA, each one equipped with a four-hydrophone array which can record both the typical acoustic signal emitted by the dolphin (social communication whistle) and the sound produced by boat engines. The permission for the deployment of the two acoustic units was issued by Italian Ministry for Infrastructures and Transports (temporary delivery of the area of the deployment to the University of Genova), Liguria Regional Agency (conservation of the regional coastal eco-system) and Ministry of Cultural Heritage and Activities (underwater prospection for possible presence of archaeological structures or remains). Moreover, the locations of the deployment were selected in agreement with the professional fishermen operating in the study area in order to avoid possible interference with their activity. The acoustic units communicate via radio link with the ground station, located inside the Portofino lighthouse. Here the raw acoustic data are processed to detect and track dolphins and boats in real time. The information continuously produced by the system is used to evaluate the level of risk connected to the detected human activity in each of the five sectors into which the study area has been divided (see Fig 1).

An alarm level is set in correspondence to the detected risk, according to the following colour code: green (no dolphins in the area), yellow (dolphins detected in the area), red (simultaneous presence of dolphins and boats in the area). The alarm level is automatically notified to the local Coast Guard that will issue presence warning messages to all people present in the area whose activity can potentially interfere with the animals. Upon reception of the messages, everybody is invited to adopt the protocol of conduct defined to reduce risks. These notifications are issued through official radio channels and several electronic message notifiers (e.g.: e-mail, SMS, mobile application).

### The study area

Though the extension of this area seems to be limited, it hosts important leisure ports (e.g. Recco, Santa Margherita Ligure, Camogli, Portono, Rapallo), industrial activities, recreational tourist areas, marine protected area. The Portofino MPA is located in correspondence of the widening of the continental shelf, which is very narrow in western Riviera and quite wide in eastern Riviera. In this area the sea current filled with water from Atlantic coming from South Tyrrhenian and Corsica Sea, turn westward creating the large gyre which is the basis of the ecosystem production in the Ligurian Sea and in Western Mediterranean. These features make the Ligurian sea an ideal feeding and breeding ground for many species of cetaceans. Recently the waters in front of Portofino promontory were recognized as core area (area highly frequented by the species) for bottlenose dolphin population [23]. Therefore the project area can be used as a model because it summarizes most of anthropic pressures as well as oceanographical and biological features. Bottlenose dolphins habitat in this area is confined within the 100 m isobaths [27], and the pleasure boat activity is confined within 3 nm from the coast line; this produces a complete overlapping with bottlenose dolphins habitat. The Ligurian coast has the highest number of docking sites (23,000 according to UCINA) in Italy; in particular close to the area of interest there are 300 docking sites in Portono, 600 in Santa Margherita Ligure, and 300 in Camogli. The assessment of boat presence in the Portofino MPA during summer 2009, as it emerges from a study conducted by Portofino MPA staff, is of about 15,000 vessels, most of which were motor boats. These data confirms that the Portofino area is subjected to a massive anthropic use leading to underwater noise pollution that is known to be a threat for cetacean. This has never been measured and/or monitored in this area and not regulated yet. The human activities drastically increase during summer when tourist boats visit the Portofino MPA and do often approach dolphins. This kind of interaction has to be regulated above all considering that newborns and calves are present in the area during summer. Therefore the area selected for system demonstration can be considered as “case study” due to the presence of a resident population of bottlenose dolphins and of a relevant part of the anthropic activities mentioned above.

### Definition of PAM system planimetry

Two acoustic units were deployed offshore, ∼1 km off the coast of Portofino headland in order to limit the sound echo effect due to the proximity of the coast. The separation distance of the acoustic units was chosen on the basis of the theoretical maximum detection range of each unit for different sea-state conditions. The calculation was made starting from the passive sonar equation, Eq (1), and imposing a lower limit to the signal-to-noise ratio at the receiver required to detect a valid source signal (bottlenose dolphin whistle) for different sea-state conditions.

$$\begin{array}{c} L_{\frac{S}{N}}\left(r\right)=SL-\left(TL\left(r\right)+NL+BW\right). \end{array}$$ (1)

Referring to Eq (1) the signal-to-noise ratio ($L_{\frac{S}{N}}$) at the receiver for a fixed range (r) is given by the difference between the source level (SL) and the sum of the transmission loss (TL) of the emitted sound through the medium and the contribution of sea-noise (NL) within the spectrum bandwidth (BW) of the source signal. This quantity must necessarily be greater than or equal to the detection threshold (DT), for a valid signal to be extracted from ambient noise, Eq (2).

$$\begin{array}{c} L_{\frac{S}{N}}\left(r\right)\geq DT. \end{array}$$ (2)

The social communication whistle emitted by bottlenose dolphins has been chosen as the source signal for target detection to the acoustic system. The bottlenose dolphin, like many other cetaceans, emits several types of acoustic signals used for specific aims. The most frequent sounds emitted are the echolocation clicks [28, 29] and the social-communication whistles [30, 31]. Unlike the echo-location clicks that are produced by many cetaceans, whistles are characteristic of dolphins and their specific spectral parameters can be used for species classification. It has been demonstrated [32] that in some cases a whistle can be associated to a specific individual so as to be defined “signature whistle”. Dolphin whistle characteristics [33] show a slight variability, mainly depending on the specific marine environment in which the dolphin lives. For Mediterranean bottlenose dolphins a typical whistle lies into 5kHz÷15kHz acoustic band with a source level of 160 dB re 1*μ*Pa @1m. According to the Shannon theory, we could have chosen a sampling frequency of about 36 kHz to have a reasonable margin of error. As will be explained in the following section, the use of cross-correlation is required for tracking. We chose *f*_*sampl*_ = 100*kHz* to gain resolution in the time domain. The calculation of the detection threshold (*DT*) starts by selecting the detection *P(D)* and false alarm *P(FA)* probabilities of the passive sonar, that in our case have been chosen as follows: *P(D)* = 0.9, *P(FA)* = 0.05. These parameters allow the extraction of the detection index (*d*) from the Receiving Operating characteristic Curves (ROC) [34] from which we obtain *d* = 9. The detection index *d* represents the gap between the mean values of the signal+noise and the noise-only probability density function (PDF). The greater the *d* value, the more we can separate signal from noise in the detection process. The detection index *d* is then proportional to the signal-to-noise ratio $\frac{S}{N_{0}}$ where *N*_0_ is the noise spectral density value calculated in the centroid of the frequency band of the signal to be detected. As can be demonstrated [34], the constant of proportionality is the number of samples *m* within the integration period of the signal considered:

$$\begin{array}{c} d=m\frac{S}{N_{0}}. \end{array}$$ (3)

where *m*, by definition, results from the ratio between the sample frequency (*f*_*sampl*_ = 100*kHz*) and the maximum frequency of the source signal to be detected (*f*_*max*_ = 15*kHz*). From Eq (3) we have

$$\begin{array}{c} \frac{S}{N_{0}}=\frac{d}{m}=\frac{9}{6.67}∼1.35. \end{array}$$ (4)

Finally, in order to be able to insert the parameter *DT* in the passive sonar equation Eq (2), we must express it as a band level (dB unit)

$$\begin{array}{c} DT=10\log\left(\frac{S}{N_{0}}\right)=10\log\left(\frac{d}{m}\right)=1.3dB. \end{array}$$ (5)

However, the value of *DT* obtained from the calculations above represents a lower limit. In fact, this value is calculated under the hypothesis of a correlation detector used in active sonar systems, where the signal envelope and frequency are known exactly, as the signal is generated by the sonar itself. In the case of a passive sonar system not only the source signal is not exactly known, but the system is even affected by secondary noise sources (multiple echoes due to coast proximity, sound reflection on the sea surface etc.). This differences can be taken into account by increasing the detection threshold (*DT*) to a safety value that is finally set to 5 dB.

The transmission loss range-dependent parameter is calculated as

$$\begin{array}{c} TL\left(r\right)=k\log_{10}\left(r\right)+\alpha r. \end{array}$$ (6)

where *r* is the source-to-receiver distance. The spreading loss constant *k* has been set to 20 considering spherical spreading for the signal in deep water (depth ∼100 m) [34] and the absorption coefficient *α*, which in general depends on frequency, has been extracted from one of the theoretical sound absorption models in seawater available in literature [35] and has been set to 0.72 $\frac{dB}{km}$, value corresponding to the centroid ($f_{c}=\sqrt{f_{min}\cdot f_{max}}$ = 8.66 kHz) frequency of the considered signal bandwidth.

For the calculation of the sea-ambient noise (NL) contribution, we made use of the Wenz curves, considered the reference model for sea-ambient noise prediction [36]. The noise spectral density value corresponding to the centroid of the frequency interval (*f*_*c*_ = 8.66 kHz) is extracted from the Wenz curves for two limit sea-state conditions: sea-state 0 (calm water) and sea-state 4 (upper limit for safe shipping).

$$\begin{array}{c} NL_{SS0}\left(f_{c}\right)=29dB\;re\;1\mu Pa. \end{array}$$ (7a)

$$\begin{array}{c} NL_{SS4}\left(f_{c}\right)=48dB\;re\;1\mu Pa. \end{array}$$ (7b)

The noise level contribution in Eqs (7a) and (7b) must be integrated over the signal frequency band by adding the term *BW* = 10log(*B*), where *B* = *f*_*max*_ − *f*_*min*_ is the signal bandwidth and corresponds to 10^4^ Hertz. We have

$$\begin{array}{c} NL_{SS0}=NL_{SS0}\left(f_{c}\right)+10\log\left(B\right)=29dB+40dB=69dB \end{array}$$ (8a)

$$\begin{array}{c} NL_{SS4}=NL_{SS4}\left(f_{c}\right)+10\log\left(B\right)=48dB+40dB=88dB \end{array}$$ (8b)

**Fig 2:**
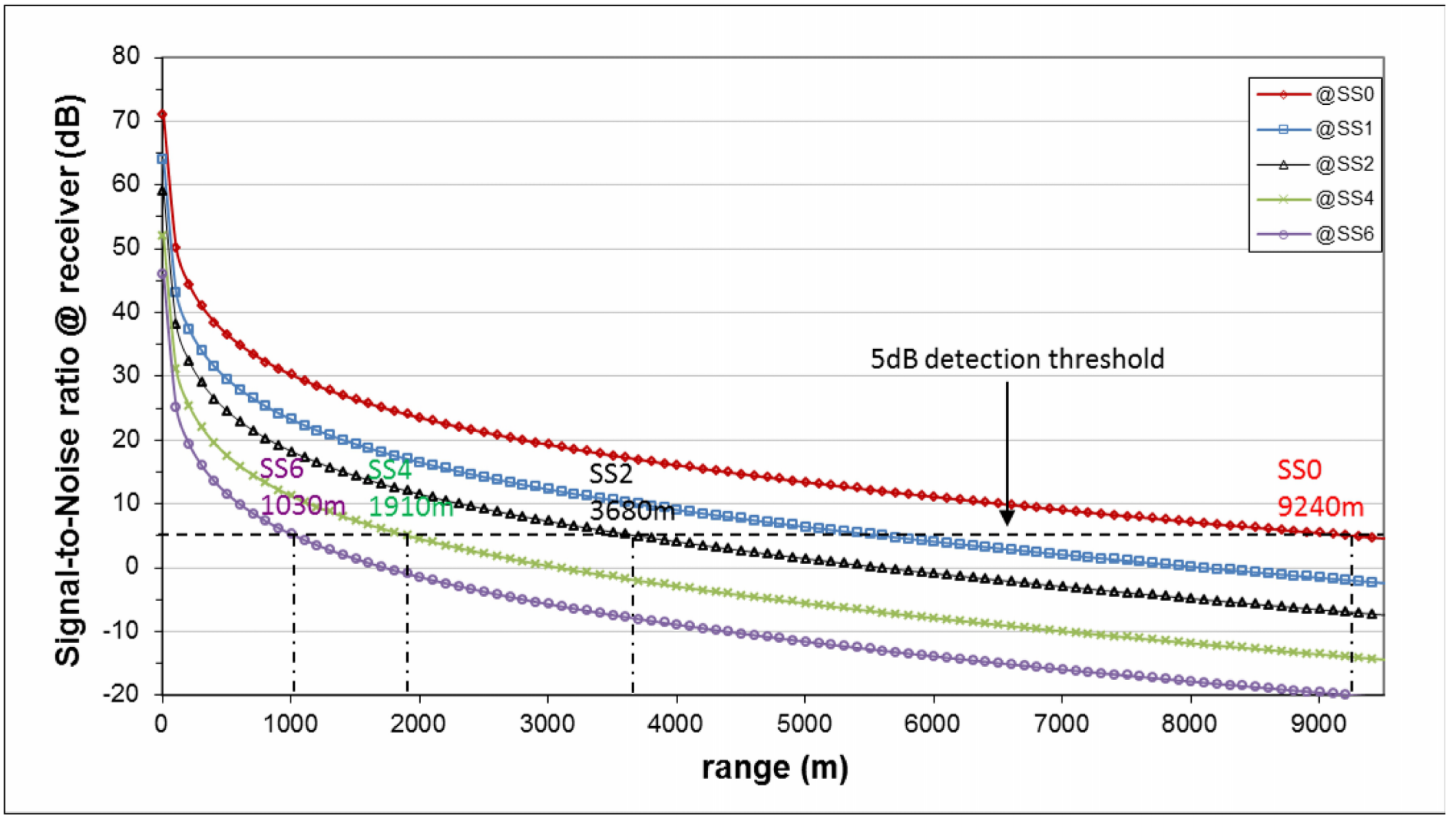
Plot of the signal-to-noise ratio level as a function of the source-to-receiver distance for different sea-state conditions. The maximum detection range of the system, for a selected sea-state, is obtained by crossing the 5 dB detection threshold (dashed line) with the corresponding signal-to-noise level curve (solid line).

Referring to Eq (2), the theoretical maximum detection range of the acoustic system as a function of the sea-state can be calculated. This can be obtained by the intersection of the 5 dB detection threshold with the range-dependent signal-to-noise ratio curves at different sea-states (see Fig 2).

The maximum predicted detection range of the system for a bottlenose dolphin whistle in the two limit sea-state conditions is then:

$$\begin{array}{c} detection\;range=\left\{\begin{array}{c} r_{SS0}∼9200m\\ r_{SS4}∼1900m \end{array}\right. \end{array}$$ (9)

However, since a general wide-band increase of sea-noise levels with respect to those resulting from the canonical Wenz curves is commonly experienced nowadays [37], a sea trial was conducted in the study area in order to acquire some ambient noise measurement. The measurement system consisted on a low-noise calibrated digital hydrophone deployed from a rigid-hull inflatable boat at about 17 meters depth, a receiver and a laptop to record data for off-line post-processing. The results of data analysis showed a measured sea-noise level corresponding to sea-state 3-4, about 7 dB greater than the one experienced on the boat (sea-state 2-3). We decided to use this scale factor to reset the maximum detection range of the acoustic system for the limit sea-state conditions, considering sea-state 2 as our best detection condition and sea-state 5-6 as the worst. Thus the real detection range limits are

$$\begin{array}{c} detection\;range=\left\{\begin{array}{c} r_{SS0}^{\prime }∼3700m\\ r_{SS4}^{\prime }∼1500m \end{array}\right. \end{array}$$ (10)

On the basis of the above calculations, the separation distance of the acoustic units was set at 1.8 km. For identification purpose the two units are referred to with the name of the headland localities they face: *Casa Sindaco* and *Punta Carega*.

### The acoustic unit

**Fig 3:**
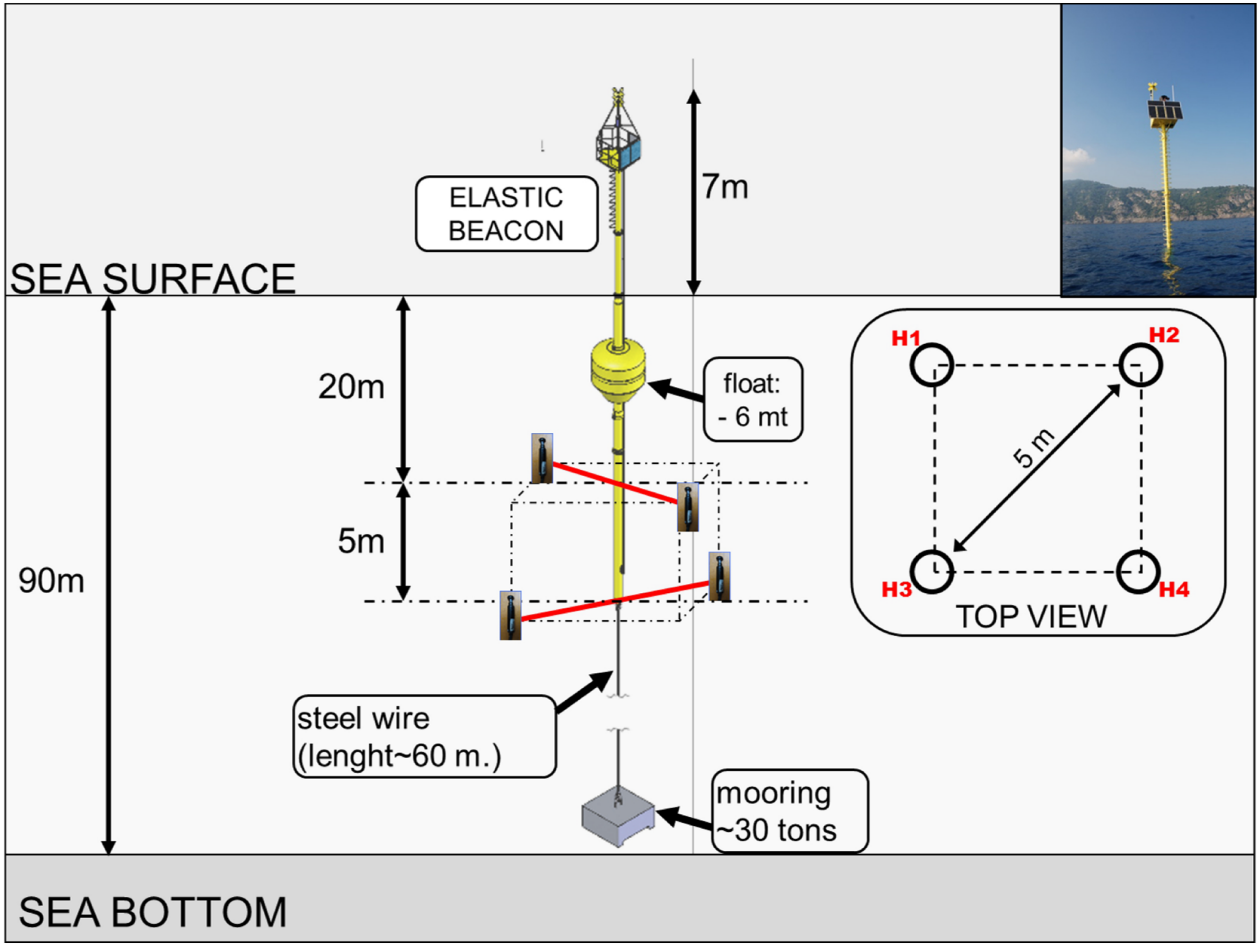
Scheme of the acoustic unit.

The acoustic unit represents the fundamental component of the whole PAM system. It consists of a particular type of bottom-moored, solar-powered marine floating structure, named “meda elastica” (elastic beacon), equipped with a 4-sensor array in its submerged part and a control turret on top of the emerged part. Fig 3 reports a scheme of the acoustic unit.

The structure is composed of a main pole, about 30 meters length, emerging for 7 meters. The top control turret hosts a watertight box containing all the electronic devices aimed to the digitization of the acoustic signals received at the four sensors, the radio broadcasting of digital data to the ground station and the acquisition of some auxiliary parameters involved in the tracking algorithms. The turret also hosts the power system of the acoustic unit, consisting on a group of six photovoltaic modules (ET MODULE, 95W_*p*_, 0.62 m^2^) mounted upon a 30 degree-tilted mechanical support and a 600 Ah, 12V battery package (Haze HZB12-100), connected to the PV modules via a remotely-monitored charge control device. The power system has been dimensioned to be able to fulfil the instant power consumption of the acoustic unit (∼35W) even in case of 4-5 consecutive no-light days due to bad weather conditions, especially during winter. The bottom side of the pole is hooked to a 60-meter length anti-twist steel wire ending with the mooring element, a 30 tons concrete block laid on the sea-floor (∼90 meters depth). The floating element is positioned at about 6 meters below the sea level and it provides the upward push to maintain tension on the whole structure and a high degree of stability even in case of sea-storm. The 4-sensor array is positioned at about 20 meters depth and it is arranged in a particular geometrical configuration: two orthogonal pairs at different depths. The hydrophones of each pair, 5 meters spaced, are positioned at the end of stiff iron arms, solidal to the main pole. The difference in depth of the two hydrophone pairs is 5 meters and their relative orientation (orthogonal) is fixed. The geometry of the array, though implying more complex tracking algorithms, is the most suitable for mechanical constraints as it allows to have at least three hydrophones always free from shadowing by any mechanical frame of the structure. The acoustic sensors are COLMAR GP0280-M omnidirectional hydrophones with a receiving sensitivity of −169 *dB* *re* 1*V*/*μPa*; −3dB: 740 Hz to 68 kHz. The acoustic signals received at the hydrophones are driven from underwater to the watertight box hosted inside the control turret of the unit and containing the electronic devices for signal processing. The analog signals are first bandpass filtered (−3 dB: 3 kHz to 23 kHz) to suppress low-frequency sea-ambient noise and high-frequency aliasing effect, then amplified (4-value remotely selectable gain:16-36-56-76 dB) and finally sampled by a 4-channel, 100 kS/s, 16 bit, simultaneous sampling ADC module, housed into the onboard reconfigurable control/acquisition unit by National Instruments, named CompactRIO. The digitized signals are transferred through an ethernet link to the TOWNET 200-20-BR26 Hyperlan MultiCPE wi-fi antenna (5.470-5.725 GHz—72 Mb/s max bandwith) mounted on top of the control turret. Data are transmitted through a transparent wireless bridge to the corresponding receiving antennas located at the ground station (Portofino lighthouse), about 4 km off. The term transparent wireless bridge refers to a wireless connection between two hosts that operates in a way that is transparent to both the network’s connected hosts. This type of connection doesn’t make use of devices devoted to data traffic routing (routers and switches), hence the term “transparent”, thus accelerating the data transmission. In addition to the hydrophone signals, auxiliary data are continuously sampled by some specific devices located onboard the acoustic units and sent to the ground station. According to their specific low-cadence acquisition rate, they have been labeled as “slow control” data. These devices all communicate with the onboard control unit (CompactRIO) via RS232/422 protocol, thanks to specific serial 4-port modules housed into the 8-slot CompactRIO Chassis. Each acoustic unit is equipped with the following components:

- OceanServer OS5000 3-axis tilt-compass device: it is mounted on the top part of the box and guarantees continuous acquisition of the real time attitude of the acoustic unit (pitch, roll, compass) necessary for the tracking algorithms.
- GPS device (SEA cRIO GPS mobile module): communicates with CompactRIO for the acquisition of the real time absolute geographic position and time synchronization of the acoustic units.
- RF solutions Protext-din GSM 2-channel remote switch. This device allows to perform a remote power reboot of the whole onboard devices in case of hardware or software crash by simply sending a text message and allows to perform a remote monitoring of the power status of the acoustic unit by automatic text messages in case of given failure condition.

One of the acoustic units (“Casa del Sindaco”) has been equipped with a high precision remote-controlled marine-meteo station on the basis of an agreement signed with the Regional Agency for Environment Protection (ARPA Liguria) and with the Department of Terrestrial-Life-Environment Science (DISTAV)—University of Genova. The data continuously recorded by the marine-meteo sensors are sent to the ground station every 30 minutes. The marine-meteo sensors, mounted onto special mechanical supports on top of the control turret are: Gill instruments WindSonic anemometer, Vaisala BAROCAP PTB330 digital barometer and Vaisala HUMICAP HMP155 humidity/temperature probe. The underwater devices, a Nortek Acoustic Wave and Current (AWAC) profiler and a Idronaut OceanSeven 316+ multiparameter water probe (salinity, conductivity, pH, oxygen level) have been deployed at about 20 and 6 meters depth respectively and fixed to specific underwater supports linked to the main pole.

### The ground station: data analysis center

Data grabbed from the hydrophones are digitized by the ADC unit and enclosed on a data stream together with “slow control” data and transmitted through a wifi bridge to the ground station.

#### Wireless data link

It is necessary to have a stable radio-link with a minimum bandwidth to transmit raw acoustic data, slow control data and timing information to the ground station. The acquisition rate, the ADC resolution, the timing accuracy and the information added by ethernet data streaming protocol set a minimum for the required bandwidth. If considering a sample rate of 100 KS/s on 4 channels with 16 bit digital output resolution the minimum required bandwidth is about 6.4 Mbit/s. Taking into account ethernet streaming extra information, a more realistic value is about 8 Mbit/s. Many wireless devices (e.g. cordless phones, garage door openers and other home appliances) commonly use the 2.4GHz frequency range. An Ethernet bridge can take advantage of the reduced noise, increased number of channels and newer technologies available for devices operating in the 5GHz zone of the spectrum or eventually use dual band mode to increase the number of available channels. At these frequencies, especially for 5Ghz, obstacles does not allow the link thus a first physical limit to the maximum achievable range come from LOS (Line Of Sight) condition (*R* + *h*)^2^ = *D*^2^ + *R*^2^. This equation gives the distance of the horizon (D) we see from an altitude *h* given the radius of the Earth *R*. If *h* ≪ *R* we may write *D*^2^ ≃ 2*Rh* and estimate $D=\sqrt{2Rh}$ for *R* = 6371*km*. The height of Portofino lighthouse (where we installed our antennas) is about 40 m giving a theoretical limit of about 22 Km. Moreover the radio frequency LOS should be kept free from obstacles in the Fresnel zone in order to minimize the loss of efficiency of data transmission caused by reflection and absorption. To achieve this result about 60% of the radius of the first Fresnel zone should be free from obstacles. Given the radius (in meters) of the first Fresnel zone $r=17.31\sqrt{\frac{d_{1}d_{2}}{fd}}$ where *d*_1_ and *d*_2_ are distances from an obstacle to the link point, *d* is the total link distance in kilometers and *f* is the frequency in GHz, if we consider the radius at its maximum ($d_{1}=d_{2}=\frac{d}{2}$) we find *r* ≃ 5*m* for “Casa Sindaco” unit (*d* ≃ 1.7*km*) and *r* ≃ 7*m* for “Punta Carega” unit (*d* ≃ 3.5*km*) corresponding to the optimum height for antenna installation. Thus, if considering that the transmitting antennas are mounted on top of the control turrets (7 meters height), we are in good operative conditions.

#### Link budget calculation

The link budget equation takes into account every component of the whole data link path, starting from the transmitter TX to the receiver RX.

$$\begin{array}{c} P_{TX}-C_{LS}+G_{TX}-L_{FS}+G_{RX}-C_{LS}=P_{RX} \end{array}$$ (11)

where *P*_*TX*_ is the transmitter power, *C*_*LS*_ is the cable loss, *G*_*TX*_ is the transmitter antenna gain, *L*_*FS*_ is free space loss, *G*_*RX*_ is the receiver antenna gain and *P*_*RX*_ is the resultant power at the receiver. To achieve a stable transmission link the condition *P*_*RX*_ > *RXsensitivity* (receiver sensitivity) must be satisfied and a reasonable link budget margin of at least 14dB to 22dB should be provided, in both direction. We need bidirectional transmission to eventually send new programs to the CRIO device installed on the buoys. The equation for free space loss is

$$\begin{array}{c} L_{FS}=32.45+20\log\left(D\right)+20\log\left(f\right) \end{array}$$ (12)

where *L*_*FS*_ is expressed in dB, *D* is expressed in km and *f* is expressed in MHz. According to this equation, we estimated a loss of about 111 dB for “Casa Sindaco” unit and 117 dB for “Punta Carega” unit. On the basis of the above calculations we chose two couples of townet 200-20-BR-26 antennas in bridge with a margin of about 44 dB and a bandwidth of up to 70 Mbit/s. The main problem we have to face is buoy own-axis rotation, mainly due to strong underwater current flowing in the area, which is much higher than expected from simulation models (even more than 200deg) and may cause connection drop down affecting the system uptime, especially for “Casa Sindaco” unit.

#### Data flow

**Fig 4:**
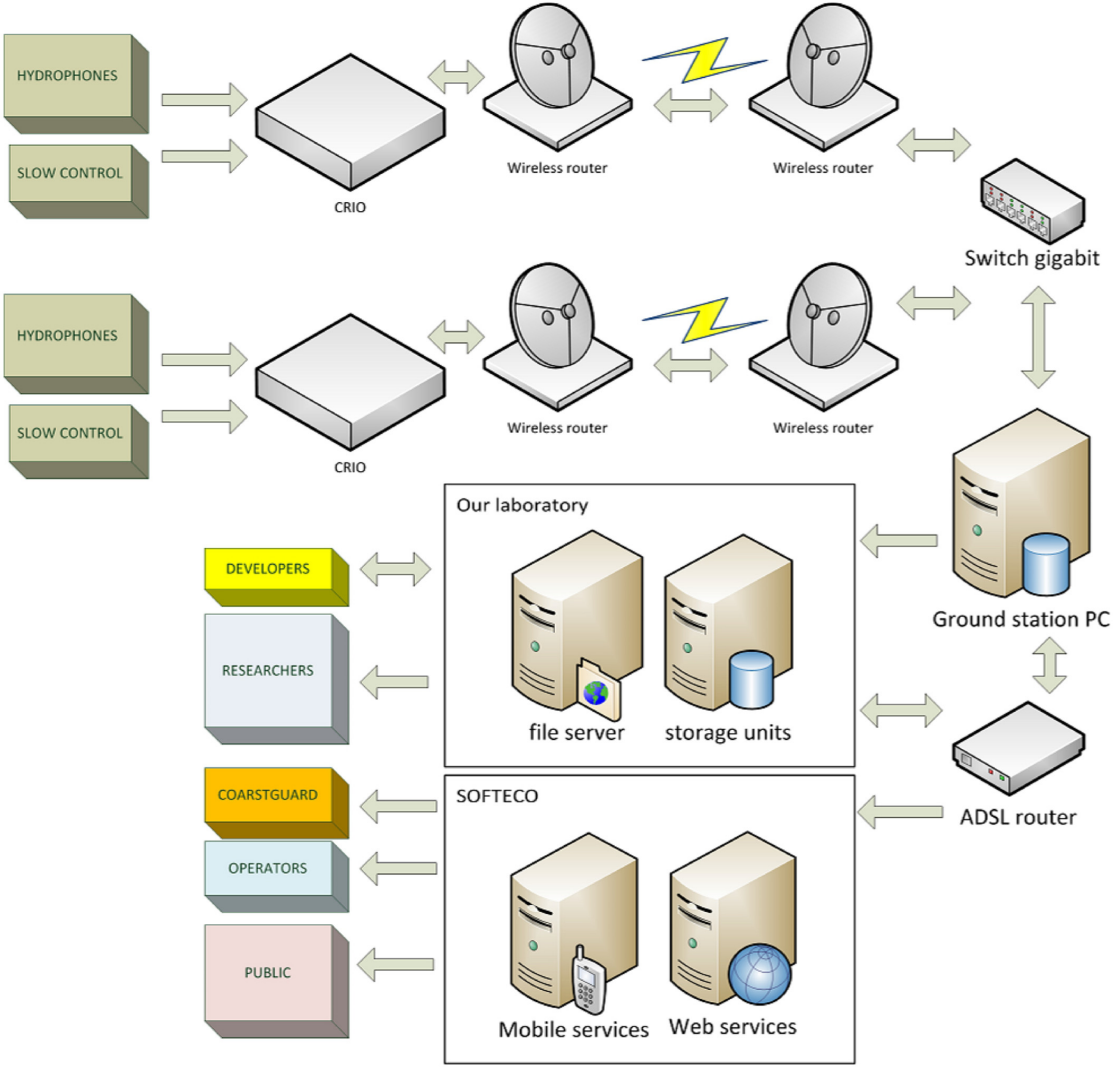
Scheme of the data flow.

A gigabit Ethernet switch connects the two bridges to a PC running the analysis software. The software generates a set of data files containing raw data and controls. Every raw data file contains 3600 s of samples from 4 hydrophones, timing control and auxiliary information in a.tdms (labview proprietary) format with a fixed size of 5.8 GByte. Slow control data are stored on auxiliary.tdms files to simplify further analysis. Every 6 hours each file on the first buffer, for every buoy, is compressed and stored on a secondary buffer unit, if enough space is available. This process runs in the background. If this operation ends correctly the first buffer is flushed. Every 24 hours another running process moves the compressed files from the secondary buffer to an USB3 HD. If the external storage unit is full or disconnected, the raw data are temporarily stored in the secondary buffer for about 2 months until the external drive is emptied or reconnected by human operators. Every time the presence of any file is checked and any file eventually not present is migrated. Every week the files archived on the external unit are removed from the secondary buffer. Apart from this mechanism a server downloads every 5 minutes the results of the analysis procedure running on the PC and publishes it on a private site accessible to the researchers. They can optionally download the raw data file if writing operations on this file ended or monitor the acquisition if not. Slow control data are downloaded every six hours for monitoring purposes and auxiliary analysis. Data are stored on dedicated on 20Tb storage units for further analysis. A similar mechanism allows the asynchronous uploading from the analysis PC (lighthouse) directly to a transit area on SOFTECO Sismat (local IT company involved in the project partnership) servers. From this area SOFTECO software generates the detection warning messages and updates for any web service in operation. Every 24 hours another program running in the background extracts raw data from the .tdms file and generate .wav files to make acoustic data available to other research groups. A scheme of the data flow is reported in Fig 4.

### Target detection and tracking algorithms

**Fig 5:**
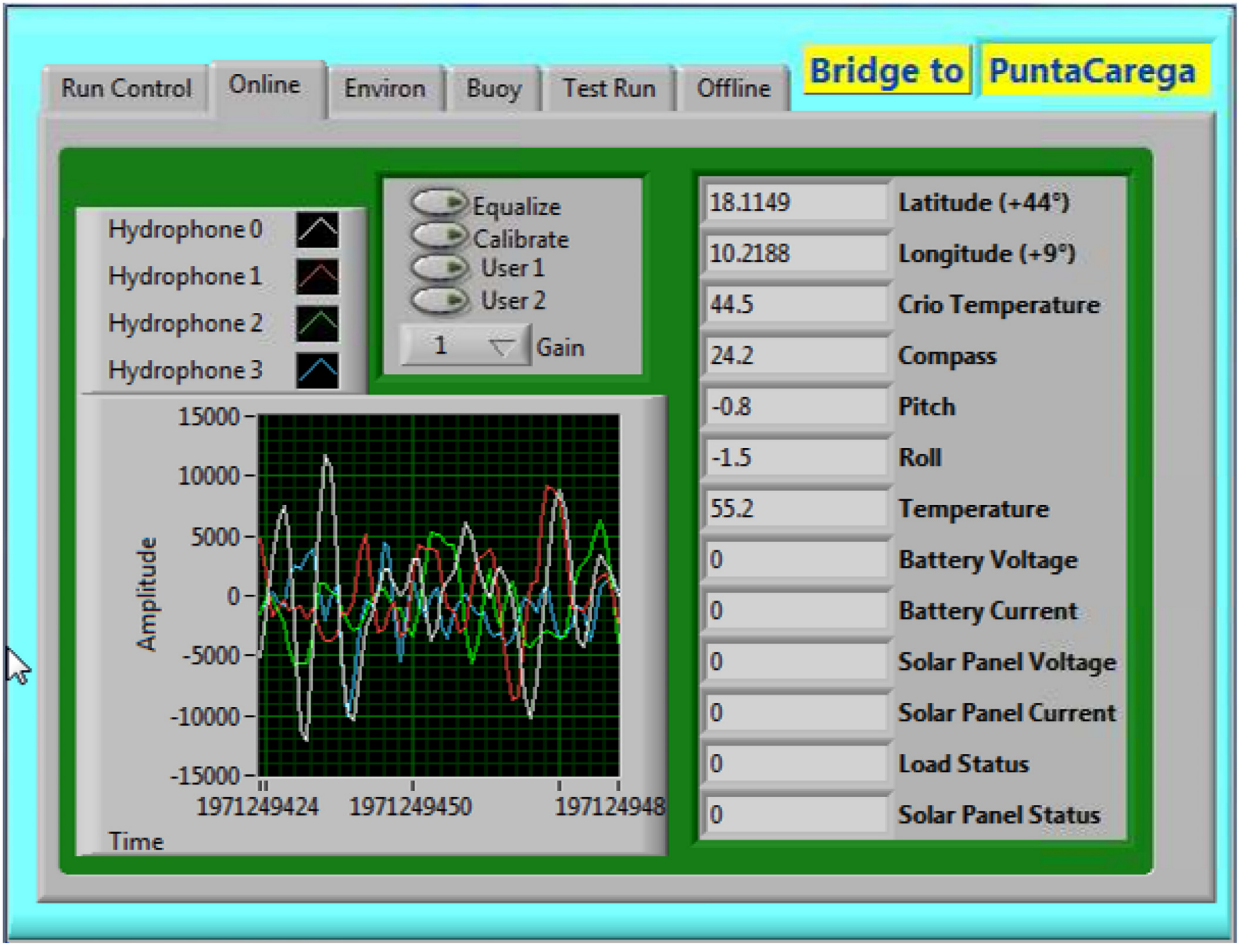
The online panel.

The acoustic data collected by the sensor array are processed in real time by a custom-made LabView (National Instruments) program. The complex architecture of the software can be roughly divided into two parts. The first part resides and runs on the CompactRIO/fpga onboard controller and it is dedicated to the acquisition, digitization and wi-fi transmission of the underwater acoustic signals received at the hydrophones and of the mentioned “slow control” parameters regarding the buoy status. Dealing with real time data acquisition, fast command execution and efficient data exchange are required, thus this portion of software is optimized for the CompactRIO native OS and it resides on the flash RAM of the device. As an example of the graphical user interface of the LabView software, a snapshot of the online panel, is reported in Fig 5.

In this panel, the four acoustic tracks and the status parameters of each acoustic unit are displayed in real time. Via this panel, the user can also modify some acquisition parameters (amplification gain, sea ambient noise equalization, hydrophone calibration signal) by remotely interacting with the onboard custom-made filter-amplifier card. Collected data are transmitted upon transparent wireless bridge without affecting cRIO performances. The second part of the software runs on the master PC located at the ground station and it is responsible for the proper data analysis, local storage of the raw data stream received from the sea and delivery of the analysis results to the entities involved in the project. The core of this portion of software consists of the detection and tracking algorithms that start from the raw data processing to obtain the real time detection and tracking of the acoustic targets within the study area. The acoustic target direction is calculated separately by each acoustic unit and each detection event is time-tagged by the onboard GPS devices. The real time position of the target is then obtained by triangulation method. The algorithms implemented in the data analysis software can be divided into three main stages: whistle detection, dolphin tracking and boat tracking.

#### Whistle detection

**Fig 6:**
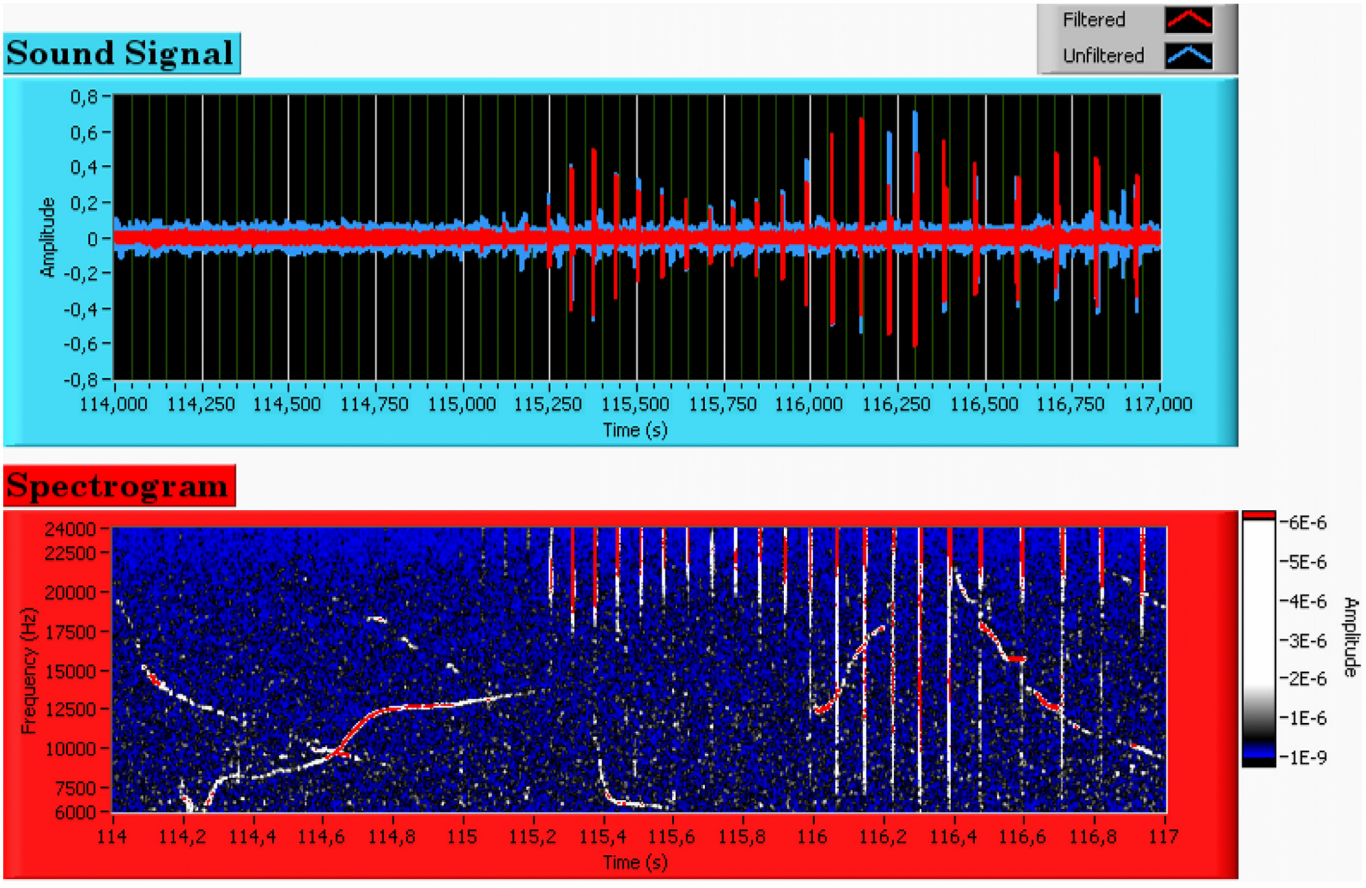
Time series (top) and spectrogram (bottom) of multiple bottlenose dolphin whistles recorded next to the Tuscany coast in May 2012.

Dolphin whistles are extremely variable in both duration and frequency and appear with such a variety as to challenge classical matched filter [38] signal detection approaches. As an example, let us consider Fig 6 where we see the time series (top) and the corresponding spectrogram (bottom) of mixed bottlenose dolphin whistles and clicks.

**Fig 7:**
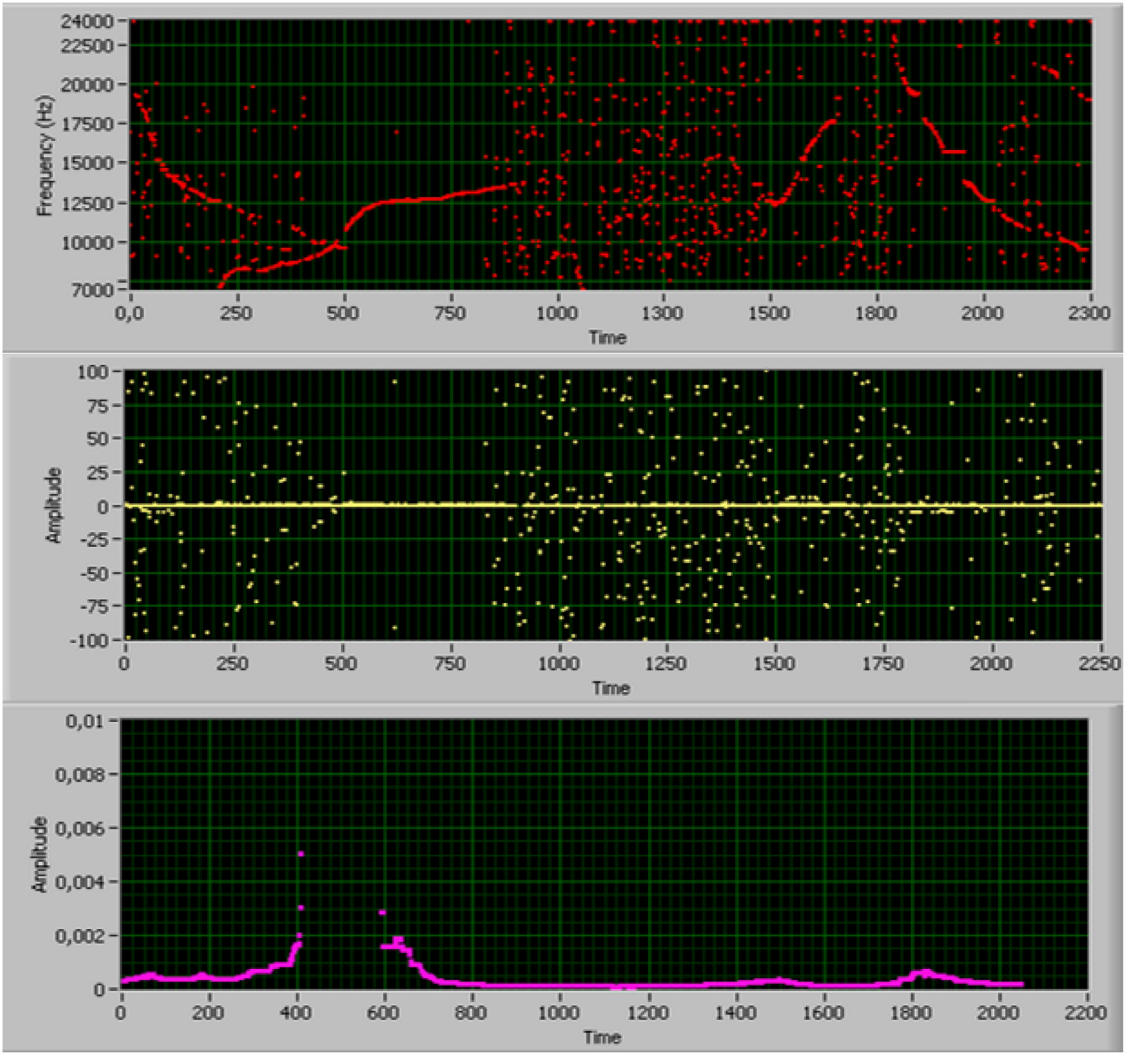
Steps of the whistle detection algorithm. Top: binary spectrogram resulting from click suppression. Middle: first derivative of the binary spectrogram. Bottom: boolean whistle detection function.

It is clear that clicks are easier to see in the time series, whereas whistles are more obvious in the spectrogram display. As a matter of simplification, whistles are usually only described by the frequency function and the development of a whistle detector will most likely be based on the spectrogram. For this purpose, raw input waveforms are processed (1024-point FFT, hanning window, 75% overlap) to obtain the real time spectrogram of the acoustic signal received at the hydrophones. The clicks are wide band impulsive signals that often superimpose on whistles and they can be considered as noise from the whistle detection point of view. Thus, as very first step of the whistle detection algorithm, data are pre-processed in order to remove undesired wide band signals from the spectrum. Referring to the spectrogram in Fig 6 (bottom), a suitable approach in whistle detection is to detect tonal peaks in the spectrum for each time bin. According to [39]- sec.4.4 we use the negative second derivative of the spectrum Eq (13) as indicator of the local maxima to transform the equalized spectrogram into a form that is suited for threshold detection.

$$\begin{array}{c} D_{i}\left(f\right)=2P_{i}\left(f\right)-\left(P_{i}\left(f-df\right)+P_{i}\left(f+df\right)\right)\;\;\;\;\text{for}\;i=1,...,N \end{array}$$ (13)

$$\begin{array}{c} M\left(i\right)=max(D_{i}\left(f\right))\;\;\;\;\text{for}\;i=1,...,N \end{array}$$ (14)

where *df* is the spectral increment of the spectrogram (y-axis), *P_*i*_* is the amplitude (z-axis) at fixed time *i* and *N* is the number of time samples in the spectrogram (x-axis). At each time step, sharp amplitude peaks (i.e. narrow-band signals) of the raw spectrogram result in local maxima of Eq (13), whereas wide-band signals (clicks) result in lower values. The absolute maximum Eq (14) at each time step (i = 1, …, N), is then selected to obtain the binary spectrogram in Fig 7 (top). As can be evinced the wide-band clicks have been successfully removed from the spectrogram.

After suppressing unwanted wide-band clicks from the spectrogram, the next step consists of building an efficient boolean dolphin whistle detector.

**Fig 8:**
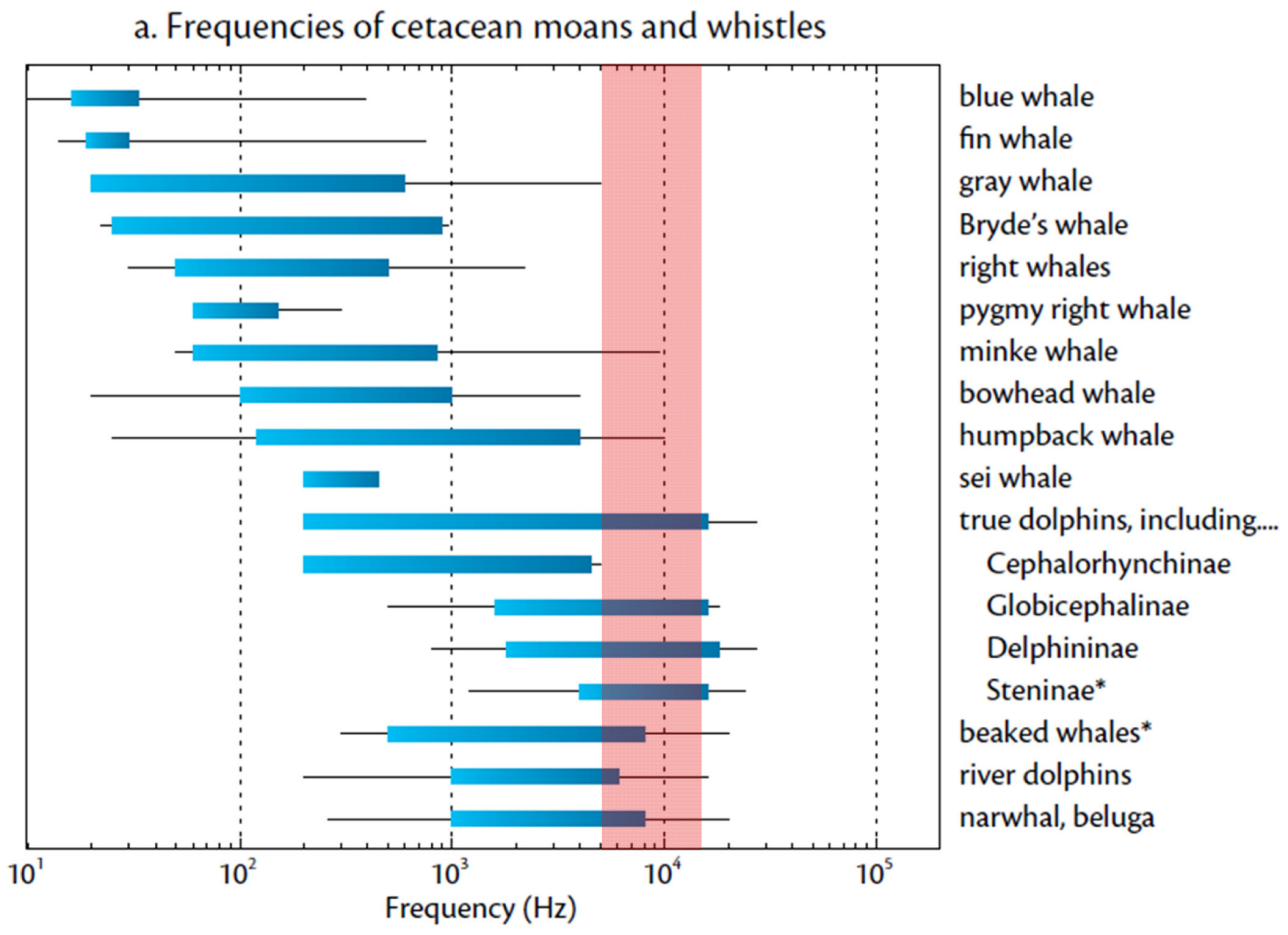
Frequencies of cetacean moans and whistles. The red band corresponds to the portion of the spectrum involved in the detection algorithm. *Reprinted from* [48] *under a CCBY license, with permission from The Oceanography Society, original copyright 2007*.

A large number of previously published papers on automated dolphin’s vocalization detection and classification is available in literature. Some works are based on the contour classification method. In [40] the discrimination between right whale upcalls and the background noise has been investigated using region-based active contour segmentation method with a resulting discrimination performance of 93%. In [41] a quantitative measure of similarity in bottlenose dolphin whistles is carried on by exploiting the contour of the fundamental frequency that is a distinctive characteristic of signature whistles. The work reported in [42] proposes a contour-based detection and classification method for the Mediterranean delphinids’ whistle, aiming at the discrimination of the whistles of five delphinids on the basis of a simple semi-automated contour analysis and of multi-variate statistical techniques. The proposed classification method achieves a global classification rate of 62.9%. A time-frequency contour extraction and classification algorithm of humpback whale vocalization is reported in [43]. The algorithm was tested on humpback whale song data recorded at various locations in Hawaii from 2002 to 2003, showing low probability of false alarm (0%–4%) under noisy environments (small vessels and snapping shrimps). Recent novel works deal with image-based whistle classification methods. An image processing technique called Local Binary Patterns (LBP) generating feature vectors for dolphin vocalization classification is proposed in [44]. A spectrogram denoising, PCA/k-means clustering classification technique of indo-pacific humpback dolphin’s vocalizations is reported in [45]. A novel approach to categorize dolphin whistles into various types is shown in [46]. A subspace of select orientation parameters of the 2D Gabor wavelet frames is used to enhance or suppress signals by their orientation, resulting in a noise-free gray-scale dolphin whistle Gabor image, which is resampled and fed into the Sparse Representation Classifier. The work reported in [47] proposes a sine-wave modeling and Bayesian inference method applied on well-defined single frequency maxima signals for automated discrimination of marine mammals vocalization from human interference. In our case, the choice of the specific whistle detection algorithm was conditioned by the real-time characteristic of the PAM system that requires time-saving and efficient computation. From this point of view, the use of image-based classification methods available from literature (more typical of off-line analysis) for the identification of the specific bottlenose dolphin whistle shape is not suitable to our case. We chose to build a preliminary version of the algorithm based on time-saving calculations that could roughly discriminate a narrow-band frequency-modulated signal within the dolphin whistle frequency band (5kHz-15kHz) of the spectrogram. A refined algorithm was then implemented on the basis of false-positive rejection emerged by the analysis of a large dataset of detections resulting from the very first months of the system activity. It is to notice that the choice of a restricted frequency range for the detector, excludes most of the cetaceans’ vocalizations from the detection candidate signals, as can be evinced from the plot in Fig 8.

Moreover, if considering the geographic position of the acoustic units (1 km off the coast) and the detection range of the system (about 4 km), only the whistles emitted by the coastal species of dolphins can be detected within the study area: tursiops (proved numerous resident population) and short-beaked common dolphins (quite rare in the Ligurian Sea). Other dolphin species as striped dolphin, long-finned pilot whale, Risso’s dolphin and Cuvier’s beaked whale live far off the coast, in correspondence of the continental slope and will hardly be detected by the system. It is clear that this approach does not exclude, in principle, to have a false-positive event contamination to the detections, but there is a high probability that a bottlenose dolphin whistle can be detected by the algorithm. On these basis, a first version of the detection algorithm was implemented and tested on a large dataset of free-ranging bottlenose dolphin whistles collected during a monitoring campaign carried on next to Pianosa island (Tuscany, Italy) in spring/summer 2011 during the starting phase of the project, obtaining good results.

Looking at the binary spectrogram in Fig 7 (top), it emerges that the time intervals containing a whistle track show a narrow distribution of maxima, whereas uncorrelated sea ambient noise is associated to a sparse distribution. This feature can be exploited for building an efficient boolean detector. For this purpose, the first derivative of the binary spectrogram M(i) with respect to time is calculated over the whole time interval:

$$\begin{array}{c} M^{\prime }\left(k\right)=M\left(k+2\right)-M\left(k\right)\;\;\;\;\text{for}\;k=1,...,i-2 \end{array}$$ (15)

The result of this operation is reported in Fig 7 (middle). As next step, the linearity of the first derivative is tested over sub-samples of size P by a detection function *W(m)*, in order to identify time intervals containing a whistle track. The detection function *W(m)* is defined as:

$$\begin{array}{c} W\left(m\right)=\prod_{j=1}^{P}w(j)\;\;\;\;\text{for}\;m=1,...,k-1 \end{array}$$ (16)

where

$$\begin{array}{c} w\left(j\right)=\left\{\begin{array}{cc} 1 & \text{if}\;|L(j)|\leq \text{threshold}\\ 0.8 & \text{if}\;|L(j)|>\text{threshold} \end{array}\right. \end{array}$$ (17)

and

$$\begin{array}{c} L\left(j\right)=M^{\prime }\left(m+j\right)-M^{\prime }\left(m+j-1\right) \end{array}$$ (18)

The function in Eq (16) increases over those time intervals characterized by small L(j) differences Eqs (17) and (18), where the first derivative of the binary spectrogram shows small deviations (whistles), and rapidly decreases when L(j) assumes greater values (sea-noise). By thresholding the detection function *W(m)*, we have a Boolean indicator for whistle detection (Fig 7-bottom).

**Fig 9:**
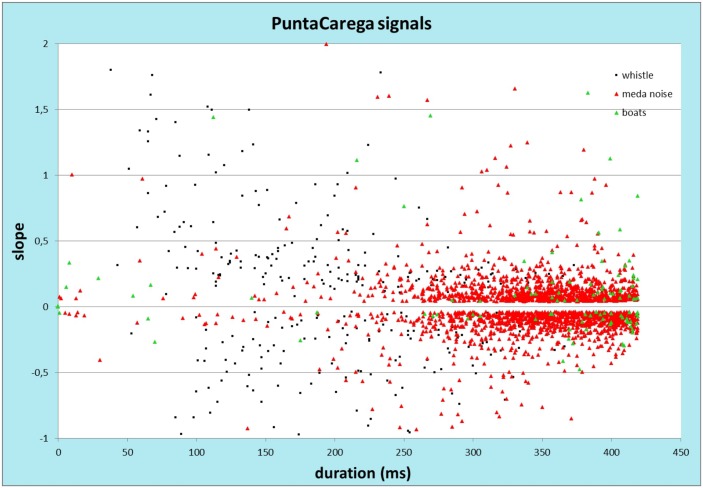
Correlation plot (frequency slope vs time duration) of 2574 detections. Whistles (black square), mooring cable (red triangle), small boats (green triangle). The origin of each signal was found by visual inspection of the corresponding spectrogram.

Once the system was deployed at sea (May 2013) and the data collection was started, the first months of recordings were used for a further test of the algorithm. From the visual analysis of a sample of 2574 mixed true/false-positive detections it emerged that the algorithm was able to detect all of the 7 main different shapes of bottlenose dolphin whistles reported in literature ([33]), thus confirming the effectiveness of the algorithm in detecting the searched signal. However, from this analysis, the whistle detection rate (DR) for the preliminary version of the algorithm resulted very low (13%). The detections were contaminated by a high rate of false-positive events mainly consisting on two kinds of artificial sounds: *(i)* sound (metal screech) produced by the torsion of the steel wire of the mooring line of the acoustic units during windy days (83%). When looking at the spectrogram this signal is very similar to a flat-type bottlenose dolphin whistle (frequency and duration); *(ii)* few specific types of small vessels (4%) When looking at the correlation plot (frequency slope vs time duration) of the detections reported in Fig 9, it emerges that the whistle signals (black square) can be separated by the artificial noise (red and green triangles) by imposing some constraints on these two variables in the detection algorithm.

The frequency slope of the track is calculated over a 52 ms (40 samples) time interval centered on the peak of the detection function W(m). Data samples out of the 3 sigma limit are discarded by the slope calculation (bisquare fit). The duration of the track is calculated as the time interval corresponding to the number of consecutive samples lying within the 25% deviation range with respect to the frequency value relative to the peak of the detection function. The choice of the boundaries of the small region of true/false-positive overlap on the correlation plot, determines the consequent true-positive loss or false-positive increase. On the basis of this analysis, an improved version of the algorithm was implemented resulting in a high false-positive suppression (8% contamination) and a corresponding rise of the whistle detection rate (DR = 92%).

#### Dolphin tracking

**Fig 10:**
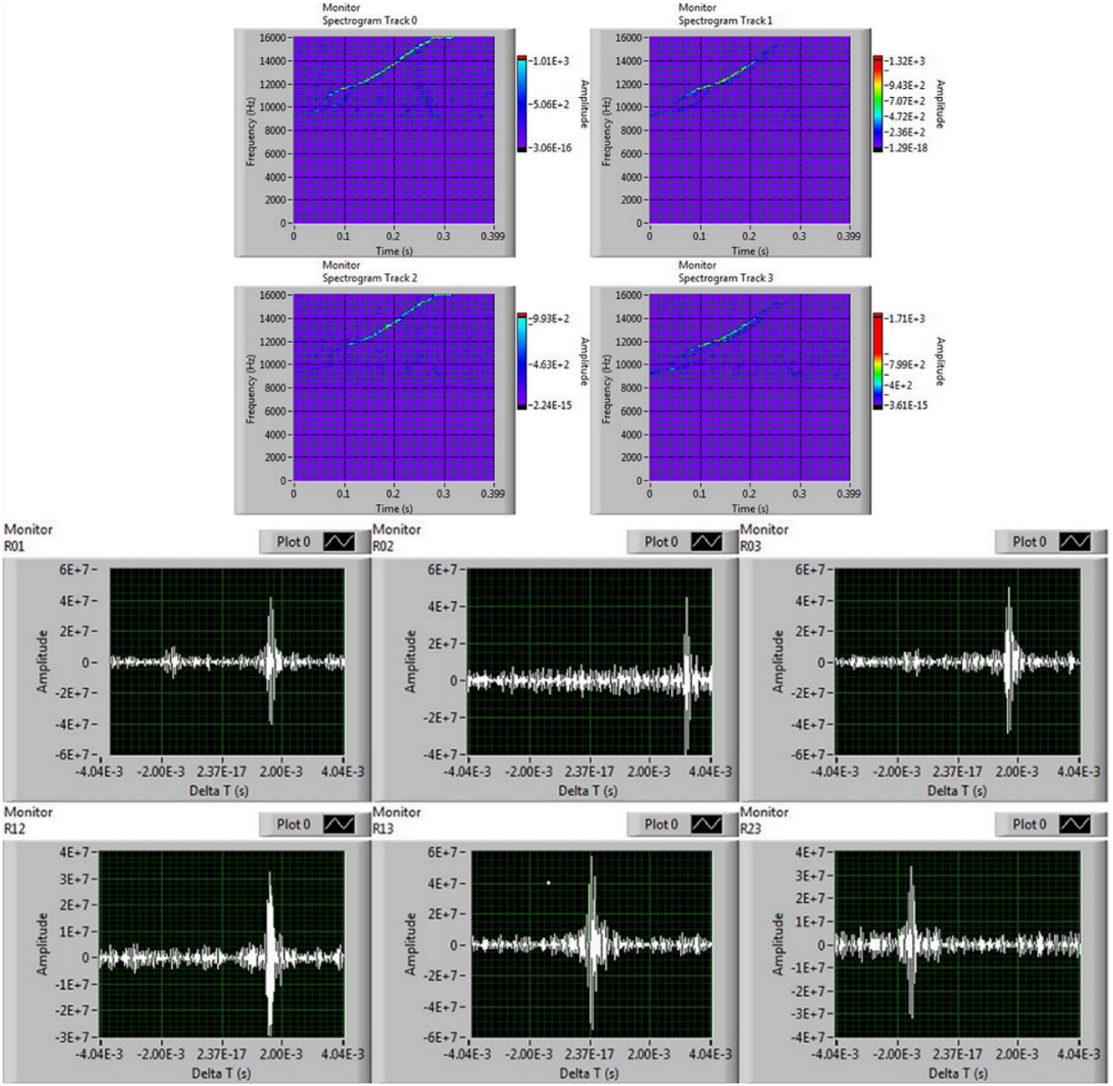
Panels extracted from the TDOA calculation routines. Top: spectrograms of a dolphin whistle at the four hydrophones. Bottom: corresponding cross-correlation plots of the six hydrophone pairs combinations. This whistle was recorded by the PAM system on October, 15th, 2013 at 02:43 a.m. (UTC).

Once the dolphin whistle has been detected, the next step of the algorithm consists of the estimation of the bearing (azimuth) and elevation angles of the acoustic source in the world coordinate system. In order to do this, the phase-gradient method, among the possible algorithms available in literature, has been adopted. This method is based on the measurement of Time Difference Of Arrival (TDOA) of the acoustic signal at different hydrophone pairs. From the point of view of the sensor array, the signal consists of an incoming plane wave propagating at incident azimuth (*φ*_*W*_) and elevation (*ϑ*_*W*_) angles, described by the wave number unit vector $\hat{n}$(*φ*_*W*_, *ϑ*_*W*_), perpendicular to the wavefront. Considering the final goal of the project that consists of detecting the presence of dolphin within the study area (range ∼ 4 km), we decided to adopt the plane wave approximation and not the spherical one to describe the acoustic signal. In fact the range within which the sound is considered as spherical wave depends on the sampling frequency and on the array geometry (∼ 100 m in this case). Beyond this limit the sound can be approximated as plane wave. This allows to use simpler and faster algorithms. If the geometry of the array is known with good precision, the measurement of TDOAs is sufficient to calculate the direction of an acoustic source in a three-dimensional coordinate system. The algorithm only uses phase information, thus allowing to ignore the wave amplitude. Triggered by whistle detection, the algorithm calculates the six TDOAs (Δt_*ij*_ for i, j = 0, 1, 2, 3) corresponding to the maximum value of the cross-correlation function of each waveform pair. The redundancy in TDOA estimation has been used for checking the consistency of the calculated target direction by crossing the information of different hydrophone pair combinations (e.g. Δt_01_+Δt_12_ = Δt_02_). As an example, the spectrograms (top) of a dolphin whistle received at the four hydrophones (recorded on October 15th, 2013 at 02:43 UTC) and the corresponding calculated cross-correlation plots (bottom), are reported in Fig 10.

**Fig 11:**
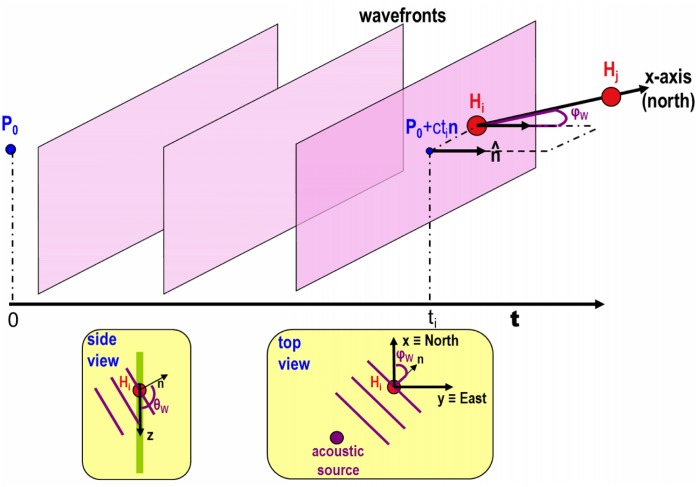
Diagram of the sound wavefront running over i-th hydrophone in the simple case of compass *ψ* = 0 and *ψ*_0_ = 0. When the wavefront reaches the sensor, it belongs to that plane. The azimuth (*φ*_W_) and elevation (*ϑ*_W_) angles of the incident acoustic wave are also shown in the diagrams at the bottom.

Since the sensor array is stiffly anchored to the buoy structure, which is not a static coordinate system, in order to calculate the absolute direction of the acoustic target, the algorithm must take into account the three-dimensional rotation of the buoy (body-fixed) coordinate system with respect to the world one. This is made possible by the tilt-compass device installed on top of the buoy which continuously records the three rotation angles (*φ*, *ϑ*, *ψ* or pitch, roll and compass) of the buoy. Moreover, the offset angle *ψ*_0_ (see section), corresponding to the absolute orientation of the sensor array with respect to the compass indicator for each acoustic unit, must be known to completely define the absolute direction of the acoustic target. Assuming to be in the plane wave approximation, the wavefront of a plane wave is defined as the set of points belonging to a plane that is perpendicular to the wave propagation unit vector $\hat{n}$ at time *t* and the wavefront reaches the i-*th* hydrophone H_*i*_ at time *t_*i*_* when it belongs to the wavefront. Referring to the scheme in Fig 11 this condition can be expressed as:

$$\begin{array}{c} \left(\vec{H_{i}}-\left(\vec{P_{0}}+ct_{i}\hat{n}\right)\right)\cdot \hat{n}=0 \end{array}$$ (19)

where

$$\begin{array}{c} \hat{n}=\left(\begin{array}{c} u\\ v\\ w \end{array}\right)=\left(\begin{array}{c} \sin\vartheta_{W}\cos\varphi_{W}\\ \sin\vartheta_{W}\sin\varphi_{W}\\ \cos\vartheta_{W} \end{array}\right) \end{array}$$ (20)

is the plane wave versor. The unknown $\vec{P_{0}}$ can be removed by subtracting different pairs *i-j* of members of the equation system Eq (21a). Three equations are sufficient to solve the system for the three uknowns *u, v, w*.

$$\begin{array}{c} \left(\vec{H_{i}}\cdot \hat{n}-\vec{P_{0}}\cdot \hat{n}+ct_{i}\right)-\left(\vec{H_{j}}\cdot \hat{n}-\vec{P_{0}}\cdot \hat{n}+ct_{j}\right)=0. \end{array}$$ (21a)

$$\begin{array}{c} \left(\vec{H_{i}}-\vec{H_{j}}\right)\cdot \hat{n}-c\left(t_{i}-t_{j}\right)=0. \end{array}$$ (21b)

$$\begin{array}{c} u\Delta H_{x}^{ij}+v\Delta H_{y}^{ij}+w\Delta H_{z}^{ij}=c\Delta t_{ij} \end{array}$$ (21c)

where Δ*t*_*ij*_ is the measured TDOA between *i-th* and *j-th* hydrophone (i, j = 0, 1, 2, 3) and *c* is the underwater sound speed. A possible choice is to consider couples of contiguous hydrophones (*H*_0_*H*_1_, *H*_1_*H*_2_, *H*_2_*H*_3_) and solving the linear system in Eq (22) for the three unknowns *u, v, w*, components of the vector unit $\hat{n}$.

$$\begin{array}{c} \left\{\begin{array}{c} u\Delta H_{x}^{01}+v\Delta H_{y}^{01}+w\Delta H_{z}^{01}=c\Delta t_{01}\\ u\Delta H_{x}^{12}+v\Delta H_{y}^{12}+w\Delta H_{z}^{12}=c\Delta t_{12}\\ u\Delta H_{x}^{23}+v\Delta H_{y}^{23}+w\Delta H_{z}^{23}=c\Delta t_{23} \end{array}\right.\; \end{array}$$ (22)

The azimuth and elevation angles *ϑ*_*W*_ and *φ*_*W*_ of the sound wave emitted by the acoustic target can be calculated by inverting Eq (20).

It is to notice that the quantity Δ*t*_*ij*_ has been extracted from the cross-correlation method and it is affected by experimental error. Let’s call it $\Delta t_{ij}^{meas}$. When inserted into Eq (21c), it could lead to ambiguous solution of the linear system. In order to avoid this problem and get real values for azimuth and elevation angles, the non-linear least square method is usually adopted. The TDOAs at the hydrophone pairs are calculated for all possible values of (*φ*_*W*_, *θ*_*W*_) within space as expected from theory, thus yielding $\Delta t_{ij}^{theo}(\varphi_{W},\theta_{W})$ values. Then, the quantity

$$\begin{array}{c} G\left(\varphi_{W},\theta_{W}\right)=\sum_{i,j=0}^{3}{\left[\Delta t_{ij}^{meas}-\Delta t_{ij}^{theo}\left(\varphi_{W},\theta_{W}\right)\right]}^{2} \end{array}$$ (23)

is calculated, and the minimum of the functional *G*(*φ*_*W*_, *θ*_*W*_) in Eq (23) corresponds to the most probable value for *φ*_*W*_, *θ*_*W*_: (*φ*_*W*_, *θ*_*W*_)^*prob*^ = min[*G*(*φ*_*W*_, *θ*_*W*_)].

Finally, the azimuth and elevation angles of the acoustic source (*φ*_*S*_, *θ*_*S*_) can be obtained by the calculated angles of the propagating wave (*φ*_*W*_, *θ*_*W*_)^*prob*^ as follows: $\varphi_{S}=\left(\varphi_{W}^{prob}+\pi \right)$ and $\theta_{S}=\left(\pi -\theta_{W}^{prob}\right)$. The calculation of the acoustic target absolute direction in the world coordinate system is simultaneously performed in real time on data set received from both acoustic units and each detection event is time-tagged by the onboard GPS device. The position of the acoustic target is then determined by the triangulation method.

#### Boat tracking

**Fig 12:**
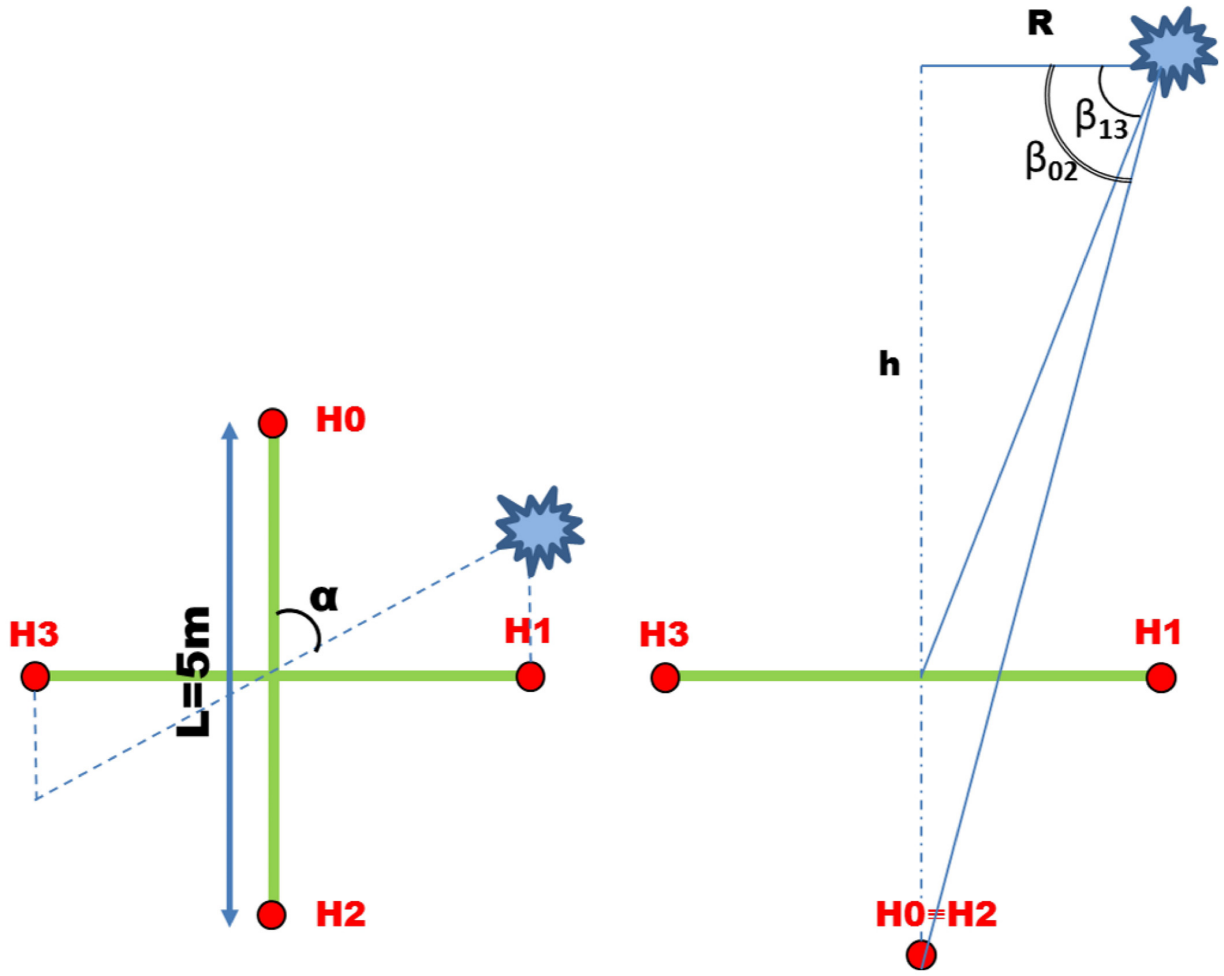
Scheme of the acoustic source position with respect to the hydrophone array. The azimuth (*α*) and elevation angles (*β*) are represented in the top view (left) and side view (right) of the scheme, respectively.

According to the main goal of the project, the presence of boats moving within the study area has to be continuously monitored in real time, in order to preserve bottlenose dolphin population from human interference and to avoid possible collisions. On the basis of the algorithm developed for dolphin tracking, a parallel process analyzes the acoustic data corresponding to the sound produced by boat engines and propellers and reconstructs the target direction as respect to each acoustic unit. The real time position of the acoustic target is then obtained by triangulation. Unlike the dolphin case, where the source signals are quite rare short-timed events, the sound produced by boat engines is a frequent event to be recorded in the study area, especially during tourist season. As a consequence, no target-detection trigger is involved in boat tracking and the process runs in non-stop listening mode thus forcing the use of lighter and faster data processing. The boat tracking algorithm is still based on the TDOA calculation by the cross-correlation method. As an example, the scheme in Fig 12(left) represents a boat, positioned at azimuth angle *α*, whose engine produces a sound signal that reaches the two hydrophone pairs (0–2 and 1–3) at different times of arrival.

**Fig 13:**
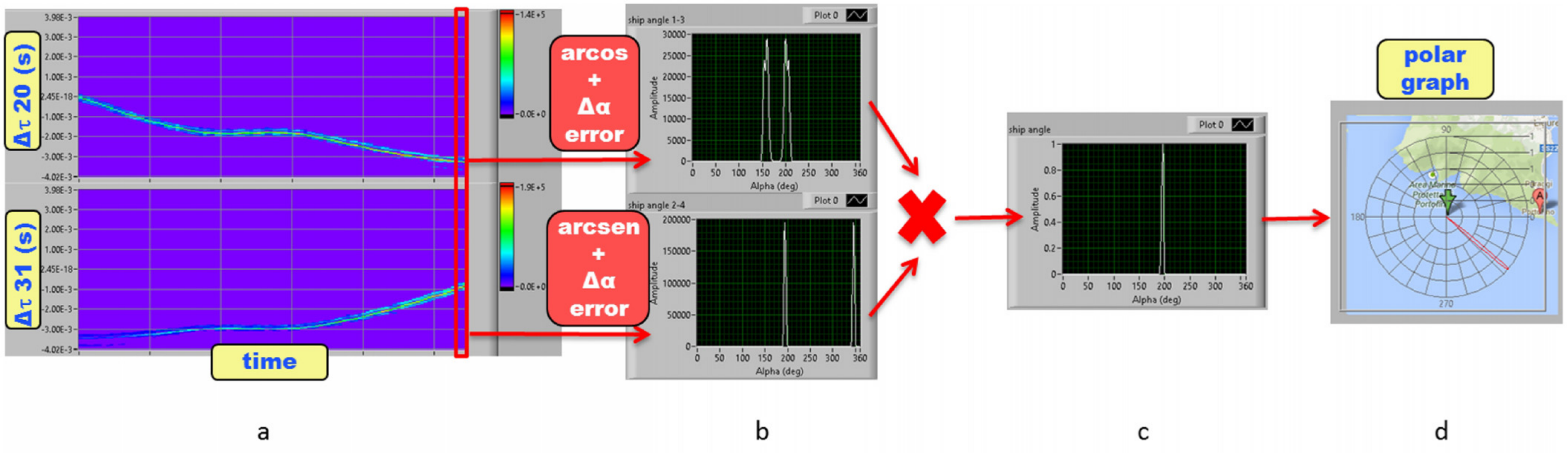
Scheme of boat tracking algorithm. (A)cross-correlogram (cross-correlation as a function of time) of each hydrophone pair. (B)probability functions of target direction associated to each hydrophone pair. (C)resulting probability function of target direction. (D)polar graph of the target direction in the world coordinate system.

Since boats are supposed to be located at sea surface, we decided to refer the calculations to the sea-surface plane. Assuming plane wave approximation and referring to the array geometry in Fig 12(left), the TDOA at each hydrophone pair is given by Eqs (24, 25)

$$\begin{array}{c} △T_{20}=T_{2}-T_{0}=\frac{L}{c}\cos\alpha \end{array}$$ (24)

$$\begin{array}{c} △T_{31}=T_{3}-T_{1}=\frac{L}{c}\sin\alpha \end{array}$$ (25)

where the quantity $\frac{L}{c}$ is the ratio between the hydrophone spacing *L* and the underwater sound velocity *c*. This parameter is crucial for tracking algorithms as it strongly depends on two quantities that have to be measured. For this reason a dedicated calibration test has been performed on field in order to determine this parameter with good precision. According to the results of the calibration test the quantity $\frac{L}{c}$ has been assumed equal to 3.43 10^−3^ *s* (see next section). The algorithm computes a 8ms cross-correlation (twice the maximum TDOA of the sound at a hydrophone pair) for both hydrophone pairs and determines the local maxima of the cross-correlation functions corresponding to the two different TDOAs △*T*_20_ and △*T*_31_. In order to suppress unwanted lower signal-to-noise ratio data samples and to avoid multiple peaks resulting from the cross-correlation of periodic signals, for each 8ms window the cross-correlation peak must exceed a user-selectable adaptive threshold related to the standard deviation of the time series distribution. If the cross-correlation maximum lies below this threshold its value is set zero. This calculation is repeated continuously, thus resulting in the corresponding cross-correlogram for each hydrophone pair (Fig 13a).

The azimuth angles *α*_20_ and *α*_31_, corresponding to the target direction to each sensor pair, are then extracted by inverting Eqs (24) and (25):

$$\begin{array}{c} \alpha_{20}=\arccos\left(\frac{△T_{20}}{\frac{L}{c}}\right) \end{array}$$ (26)

$$\begin{array}{c} \alpha_{31}=\arcsin\left(\frac{△T_{31}}{\frac{L}{c}}\right) \end{array}$$ (27)

However, the symmetry of the system with respect to the sensor array axis gives rise to an ambiguity in the determination of the target direction and the use of Eqs (26) and (27) determines a double solution (real and virtual) for the target azimuth angles *α*_20_ and *α*_31_ at each hydrophone pair. Referring to Fig 12(left) we have: $\alpha_{20}\to \alpha_{20}^{\prime }=2\pi -\alpha_{20}$ (axes 0–2) and $\alpha_{31}\to \alpha_{31}^{\prime }=\pi -\alpha_{31}$ (axes 1–3).

The ambiguity is removed by multiplying the probability functions associated to Eqs (26) and (27) at each time step. For both equations the probability function is represented by two gaussian functions centered respectively at the real and virtual angles, whose standard deviation *σ* is proportional to the measure error (Fig 13b). The measure error △*α* is obtained by the error propagation method from Eqs (26) and (27):

$$\begin{array}{c} △\alpha =\frac{1}{\sqrt{1-{\left(\frac{\tau }{L/c}\right)}^{2}}}\cdot \frac{△\tau }{L/c} \end{array}$$ (28)

where *τ* is the measured TDOA at a specific hydrophone pair and △*τ* is the time resolution of the cross-correlation. By multiplying these two probability functions, the virtual solutions (*α*′) are suppressed and the result is the real azimuth angle *α* of the target with respect to the sensor array at each time step (Fig 13c). The adopted standard deviation *σ* is calculated as 4 times △*α* to ensure the complete overlap of the gaussian functions corresponding to the real angle solutions. The accuracy of the target direction is determined by the lower *σ* value between the two couples. In particular in the case of $\tau ≃\frac{L}{c}$, *σ* is maximum for a hydrophone pair and minimum for the other.

#### Tracking system calibration

In order to validate the whole passive acoustic detection system and the corresponding tracking algorithms, an on-field calibration campaign was carried on by using an intense acoustic source of known GPS position. For the purpose, a 100-hp engine inflating boat equipped with a onboard commercial GPS device was used as acoustic target. The absolute position of the boat moving along a pre-defined pattern within the detection area was recorded continuously by the GPS device (1 Sample/s) during the calibration campaign. The two main objectives of the calibration campaign were identified as: *(i)* defining the parameter $\frac{L}{c}$; *(ii)* determining the azimuthal offset angle (*ψ*_0_) of the sensor array. The parameter $\frac{L}{c}$ (the ratio between hydrophone spacing and sound speed) was experimentally determined by analyzing the acoustic data produced by the boat engine during a series of laps around each acoustic unit at a fixed distance and collected by the four-sensor array. In this case, the measured TDOA of the sound generated by the boat engine at a specific hydrophone pair (e.g. H0-H2) as a function of time is a sin-like distribution whose local maxima (and minima) corresponds to the absolute maximum time delay at the hydrophone pair, when the boat is aligned with the hydrophones joining axis. Referring to the scheme in Fig 12(left), given the orthogonal symmetry of the sensor array, the maximum time delay for a selected hydrophone pair corresponds to the null delay for the other pair. Moreover, since the sensor array is located at a known depth below the sea level, for a correct calculation of the parameter $\frac{L}{c}$ it is necessary to take into account the elevation angle of the target boat with respect to the sensor array. Referring to Fig 12(right) the elevation angles of the boat with with respect to the two hydrophone couples can be defined as:

$$\begin{array}{c} \beta_{02}=\arctan\left(\frac{h_{02}}{R}\right) \end{array}$$ (29)

$$\begin{array}{c} \beta_{13}=\arctan\left(\frac{h_{13}}{R}\right) \end{array}$$ (30)

where *h*_02_ and *h*_13_ are respectively −20 m and −25 m and *R* is about 150 m. Then, the TDOA distribution of the sound of boat engine at each hydrophone pair can be expressed as

$$\begin{array}{c} △T_{20}=T_{2}-T_{0}=\frac{L\cdot \sin\alpha }{c}\cdot \cos\beta_{02} \end{array}$$ (31)

$$\begin{array}{c} △T_{31}=T_{3}-T_{1}=\frac{L\cdot \cos\alpha }{c}\cdot \cos\beta_{13} \end{array}$$ (32)

and the parameter $\frac{L}{c}$ is defined as: $\frac{L}{c}=\frac{△T_{ij}^{max}}{\cos\beta_{ji}}$ for the two couples. The separation distance between the boat and the acoustic unit was kept constant at about 150 meters (*β* ∼ 7°) during the calibration test, in order to keep the contribution of the elevation angle negligible (∼ 0.7%). Three laps around each acoustic unit were carried on by the target boat and the values of the corresponding local maxima of the TDOA distribution were computed by the tracking algorithms. The value of the parameter $\frac{L}{c}$ was then assumed to be the average of the detected maxima and resulted to be $\frac{L}{c}=3.43\cdot 10^{-3}$ s and $\frac{L}{c}=3.46\cdot 10^{-3}$ s respectively for *Casa Sindaco* and *Punta Carega* acoustic units. The definition of the parameter $\frac{L}{c}$ allows to solve the system in Eq (22) for the unknown vector unit $\hat{n}$ which identify the direction of the acoustic target at each acoustic unit. However, since the control turret of the acoustic units, housing the tilt-compass device, was installed after the underwater deployment of the sensor array, the overall azimuth offset angle *ψ*_0_ between the compass indicator and the buoy coordinate system fixed by the sensor array geometry is unknown. Thus, the data recorded during the calibration campaign were used to define the angle *ψ*_0_ for each acoustic unit. To this end, the position of the target-boat recorded by the onboard GPS device as a function of time was compared with the real time position calculated by the tracking algorithm for three circular trips around each acoustic unit. The difference between the two distributions calculated for each acoustic unit coincides with the overall offset angle *ψ*_0_, which results to be +30° for *Casa Sindaco* buoy and −80° for *Punta Carega* buoy.

#### Target tracking accuracy

The error associated to the target direction, especially in the case of dolphin tracking, can be hardly calculated in theory, as it depends on several contributions coming from the experimental error associated to variables involved in the tracking algorithms. A qualitative estimate can be obtained by assuming the simplest case of a target located on the sea-surface plane and considering only the azimuth angle *α* defined in the boat tracking algorithm. In order to estimate the overall error associated to the real target direction, the widths of the resulting probability functions (Fig 13c) for relevant azimuth angles extracted by gaussian fit are considered. The maximum width obtained from this analysis is assumed as the error associated to the target (boat and dolphin) direction and corresponds to 2°. Considering a target located at the limit of the detection area in calm water (distance 3700 m @SS0), this corresponds to a square error area of about 130 m side around the target position determined by triangulation.

## Results

The acoustic units were deployed in their respective positions in May 2013 and, in the next few days, they were equipped with all the acoustic and meteo-marine sensors. The passive acoustic monitoring system was switched on on May, 10th 2013. Except for some maintenance down-time periods, the system has been recording underwater acoustic data continuously, since the date of switch on. The bottlenose dolphin’s presence in the study area is continuously monitored by the local Coast Guard operators that can visualize the specific concerned sector on the map of the study area in real time. In case of simultaneous detection of dolphins and boats in the same sector, presence warning messages are sent by the Coast Guard operators to the sailors that are requested to follow the agreed protocol of conduct.

### PAM system working efficiency

In order to evaluate the efficiency in working of the PAM system two indices are calculated: the functioning rate (FR) and the bottlenose dolphins tracking rate (TR). FR was calculated as the ratio between the number of minutes in which the PAM system was active and well-functioning, and the total number of minutes in the sampling period. The sampling period to calculate FR includes 18 months, going from 10th May 2013 to 10th November 2014. According to the 18-months sampling period (790,620 mins) the calculated FR index is 56% (442,747 mins) for “Casa Sindaco” unit and 83% (656,215 mins) for “Punta Carega” unit. TR aims to evaluate the efficiency of the PAM system in tracking bottlenose dolphins once detected. TR was calculated as the ratio between the number of minutes with at least one tracked dolphin and the number of minutes with at least one detected dolphin. Since the tracking algorithm implemented in the first revision of the elaboration software was affected by a considerable number of false positive events, an improved version was implemented in the software on 7th March 2014. For this reason, the TR index calculation takes into account a period of 6 months (from March to September 2014), even if several successful tracking events were registered even before this date. If limiting to this reduced detection period we had 881 minutes with at least one detected bottlenose dolphin and 687 minutes with a bottlenose dolphin tracked by one or both acoustic units. The calculated TR index is 78% (see Table 1).

**Table 1:** Summary of the PAM system working efficiency.

	**FR**	**TR**
period of activity (min)	656,215 (442,747)	
period of sampling (min)	790,620	
period of tracking (min)		687
period of detection (min)		881
**percentage**	**83% (56%)**	**78%**

The values between round brackets refers to “Casa Sindaco” unit.

### Bottlenose dolphin tracking

**Fig 14:**
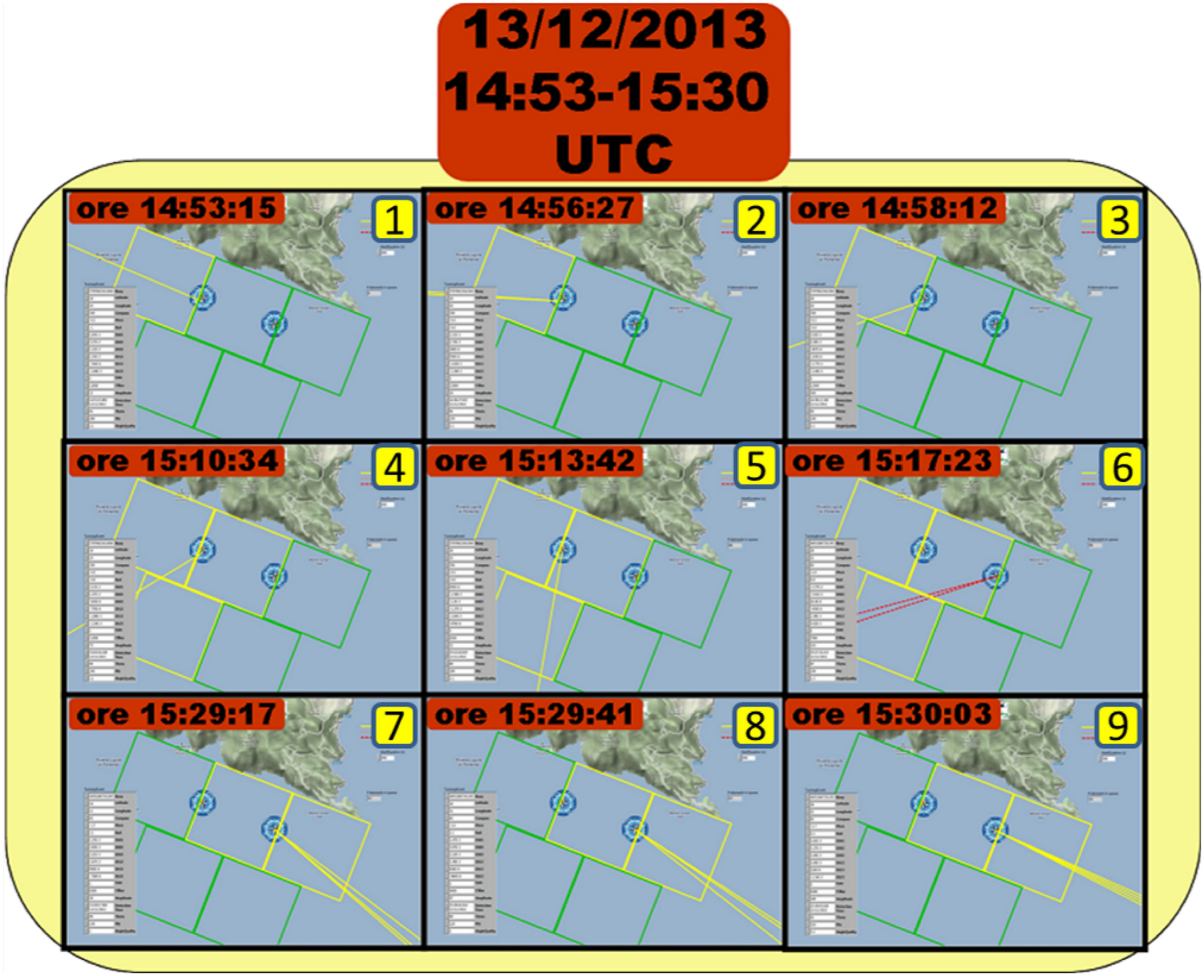
Dolphin tracking event recorded by the PAM system on December, 13th 2013. Panels from 1 to 9 show the dolphin pod direction calculated by the tracking algorithm as a function of time.

As stated, the PAM system has been designed to determine the direction of the acoustic target to each GPS-synchronized acoustic unit and find the target position by crossing the calculated directions (triangulation). The algorithm implemented for angular tracking, based on TDOA measurement by cross-correlation, have shown a high degree of efficiency. As an example, Fig 14 shows the time sequence of the angular tracking of a pod of bottlenose dolphins moving in the study area on December, 13th 2013 at 14:53 UTC.

A pod of dolphins coming from the northwest was detected by the PAM system and its direction was tracked for about forty minutes while moving towards the southeast. Each of the nine panels represents a detection event at a specific time. The pod was at first detected at 14:53 UTC (panel 1) coming from the northwest and its direction was determined only by the nearest acoustic unit (*Punta Carega*), being the target out of the maximum detection range for the second acoustic unit (*Casa Sindaco*). Two minutes later the pod had moved towards the southeast (panel 2) and kept on moving towards *Casa del Sindaco* unit (panel 3-5). After about twenty minutes since the first detection the pod was located in the middle area between the acoustic units, and at 15:13 UTC the second unit detected the pod and calculated its direction (panel 6-9) till the pod overcame the maximum detection range and was lost by the system. Unlike the angular tracking events, that have been registered with a remarkable frequency, the triangulation occurrences have been quite rare. This is probably due to the contribution of many possible causes: *(i)* the experienced whistle source level is lower than the one expected from literature; *(ii)* the whistle emitted by bottlenose dolphins is characterized by a not negligible directivity index [49]; *(iii)* the sea-noise masking effect is higher than expected.

**Fig 15:**
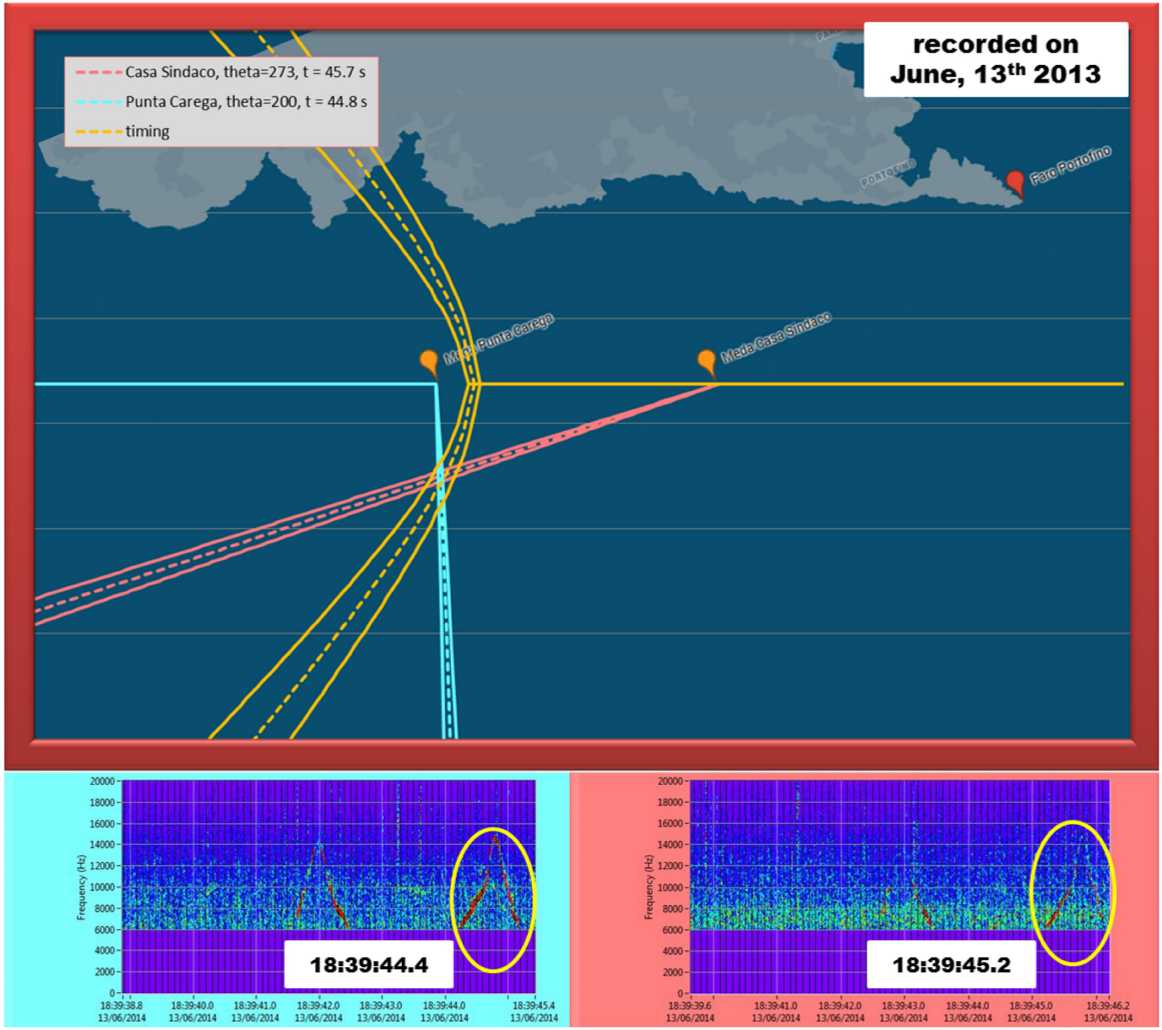
Dolphin pod localization event recorded by the PAM system on June, 13th 2013. Top: position of the dolphin pod calculated by applying triangulation to the directions (straight dashed lines) of the target to each acoustic unit. The curve dashed line represents the locus of points of equal TDOA at the acoustic units. Bottom left (right): spectrogram of the specific whistle recorded by Punta Carega (Casa Sindaco) unit for the calculation.

These factors directly affect the maximum detection range of each acoustic unit, thus reducing the ability of the system in determining the target position by triangulation. Nevertheless, some triangulation events occurred since the system switch on and a representative example, occurred on June, 13th 2013 at 18:39 UTC, is reported in Fig 15.

The radar panel (top) shows a dolphin pod detected simultaneously by both acoustic units within the study area. The straight dashed lines represent the direction of the pod with respect to each acoustic unit and the position of the pod is calculated by triangulation method. The bottom panels in Fig 15 report the spectrograms of the specific whistle detected respectively by *Punta Carega* (bottom left) and *Casa Sindaco* (bottom right) unit. The shape of the whistles detected is very similar and the time delay between the two detection events (∼ 0.8 s) is compatible with the distances involved, thus indicating that the same source signal has been detected by both acoustic units simultaneously. The curve dashed line in the top panel of Fig 15 represents the branch of hyperbole whose foci are the acoustic units. This is the locus of points of equal TDOA of the sound at the acoustic units. As can be seen, the source location identified by triangulation belongs to the branch of hyperbole and this seems to confirm the reliability of the tracking system.

### Boat tracking

**Fig 16:**
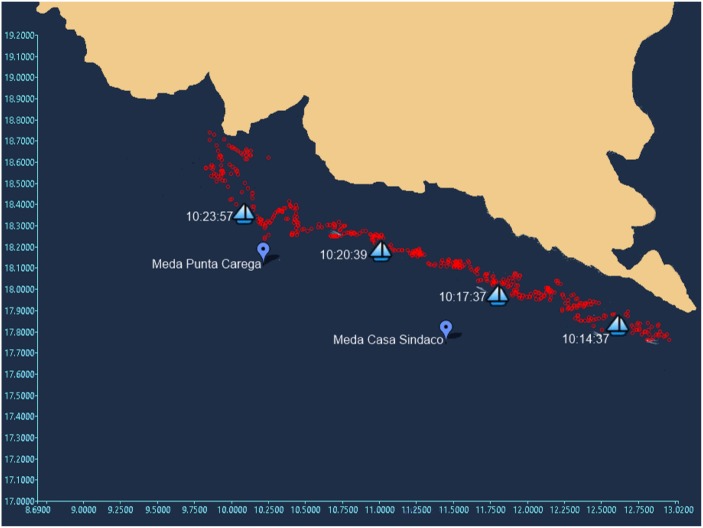
Boat tracking event recorded on March, 23rd 2015. The panel shows a 5 km route of a passenger vessel moving in the study area. The red circles correspond to the position of the boat every 1 second, as reconstructed by the PAM system. The boat-shaped placemarks correspond to the AIS-recorded position of the boat. The good correspondence confirms the efficiency of the boat tracking algorithm.

In the case of a boat, the triangulation of the target is always achievable within the detection area, since the sound produced by the boat engine is much more intense with respect to that of a dolphin whistle and this corresponds to an increase of the maximum detection range of the acoustic units. As a result the position of every boat moving in the study area is determined by the system in real time with a update rate of about 1 Hz, thus allowing the user to know the route of the target. Fig 16 shows a real case of boat tracking within the study area occurred on March, 23rd 2015.

The red circles represent the position of the boat-target every 1 seconds, calculated by the triangulation method, compared to the AIS position (boat-shaped placemarks) of a passenger vessel (https://www.marinetraffic.com). As shown above, the boat tracking algorithm seems to be efficient in tracking a single target, but it presents some deficiencies when multiple targets are present within the detection area, especially during tourist season. In this case, the cross-correlation function shows multiple peaks and, in principle, the direction of more than one target can be simultaneously calculated by each acoustic unit. However, according to the actual boat tracking algorithm, only the highest acoustic signal recorded by each acoustic unit (likely produced by the nearest boat) dominates and determines the specific target to be tracked. This can lead to the case in which each acoustic unit determines the direction of different targets, thus invalidating the triangulation method. To overcome this problem, further improvement of the boat tracking algorithm, based on the DEMON algorithm [50] aimed to the boat classification, is under study and will be tested in the next few months.

### Detection alarm generation

**Fig 17:**
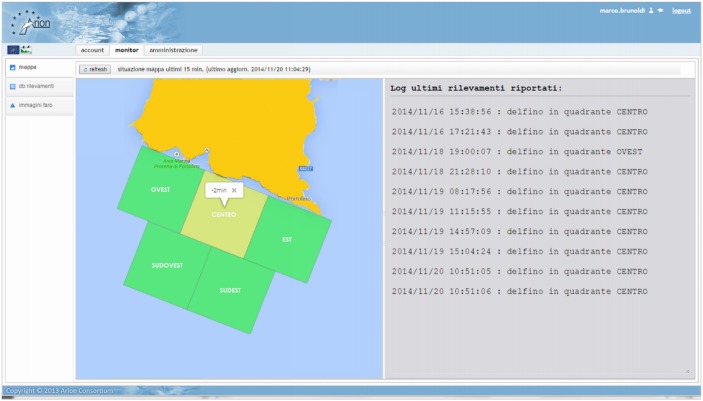
Snapshot of the alarm generation web portal. Each dolphin detection event is visualized on the map by highlighting the corresponding sector. This information is available to the local Coast Guard operators that are responsible for the alarm generation.

According to the main task of the project the stakeholders must be informed about the presence of dolphins in the study area in order to guarantee a safe coexistence between human and dolphin activities. For this purpose, dolphin detection events, elaborated at the data analysis center (Portofino lighthouse) are sent to Softeco Sismat (Genova) main web server and visualized on a dedicated web portal in real time. Fig 17 shows a snapshot of the web portal developed by Softeco Sismat.

A map of the study area, divided into 5 sectors (∼2 km side), is reported on the web page. The color of each sector is associated to a different detection status: green (no dolphin detected) or yellow (dolphin(s) detected). In case of dolphin detection, if the system notifies the simultaneous presence of a boat in the same sector, it turns to red. The detection status of the study area is constantly monitored by the local Coast Guard operators thanks to a dedicated display connected to the Softeco web server and installed at the Coast Guard headquarter. The operators are responsible for monitoring the detection status of the study area and for sending warning messages about the presence of dolphins to the sailors present in the area at the moment of the detection who have the possibility to be informed by text messages (upon registration on the official web site of the project) or via official marine VHF radio channels. Moreover, the sailors have the possibility to visualize the detection status of the area in real time by a smartphone application developed by Softeco Sismat. A HTML detection report is generated by the elaboration software and made available on the web to the scientific researchers involved in the project with a few minutes delay since the occurrence of the detection event. Every information relative to the last detection is reported on this document: date-time of detection, spectrogram of the whistle detected, a snapshot of the cross-correlation plots and the results of the tracking calculations.

## Discussion

In the frame of the European program LIFE we implemented and installed a permanent automated real-time passive acoustic monitoring system for the improvement of the conservation status of the transient and resident population of bottlenose dolphin (Tursiops truncatus) in the Portofino Marine Protected Area (MPA), Ligurian Sea. This is the first example in the Mediterranean Sea of a permanent acoustic station dedicated to the continuous monitoring of the presence of bottlenose dolphins. Two detection units have been deployed in front of the Portofino promontory and moored at a depth of 90 m. Each unit is equipped with four hydrophones and a Labview-based acquisition system, and is powered with solar panels. Hydrophone tracks are transmitted by a wi-fi dedicated connection to the ground station where they are scanned in search of dolphin whistles and then stored. The system is operative since May 2013 and it has been continuously in operation, with small dead-time, in any sea state condition (on Christmas Eve 2013 the wave height reached the record value of 10 meters without affecting the transmission rate). The detection algorithm based on [39] has been improved in order to remove the contamination due to boat and other environmental noise that could mimic the dolphin whistles. The present detection efficiency is better than 90% with a contamination of less than 1%. The hydrophones sensitivity allows to survey an area of approximately 20 km^2^ covering the whole Portofino promontory frontline. After the detection of a whistle, the localization of the tursiop is performed comparing the sound direction and the arrival time for each detection unit. Even if during the operation time several hundreds whistles have been detected and the corresponding source direction reconstructed by at least one detection unit, the probability of triangulation resulted very low (less than 1%). From the analysis of these events we have verified that the system can localize the cetacean with a precision of ≃3% at the maximum detection range (3700 m). For the scope of the ARION project, however, the two detection units operate individually because it is necessary only to locate the tursiops within one of the five sectors of the project area. The boat presence in the area has been also investigated. By comparing the reconstructed route with the GPS data taken on board the boat used for empirical calibration we have proved that the system is very precise in the tracking of individual boats in the whole project area. We are presently investigating algorithms capable to improve the reconstruction efficiency when more boats are concurrently present in the area. The detection system matched the design specification, proved to be very effective, highly reliable and with small maintenance costs and can be easily replicated in other areas where the tursiops monitoring is required.

## Supporting Information

S1 File Permission to publish Fig 8. (PDF)

S2 File Permission to publish Figs 1, 2, 3, 4, 5, 6, 7, 9, 10, 11, 12, 13, 14, 15, 16, 17. (PDF)
