# Supplementary material for: A Permanent Automated Real-Time Passive Acoustic Monitoring System for Bottlenose Dolphin Conservation in the Mediterranean Sea
Source: PLoS One. 2016 Jan 20;11(1):e0145362. doi: 10.1371/journal.pone.0145362 (PMC4720475; doi:10.1371/journal.pone.0145362)
Supplement: S1 File — (PDF) [file pone.0145362.s001.pdf]

Subject: Re: Request for permission to publish figure
From: Vicky Cullen <vcullen@whoi.edu>
Date: 17/10/2015 17:28
To: Marco Brunoldi <brunoldi@fisica.unige.it>

Thanks for your inquiry. This use is covered by the permissions statement for *Oceanography* magazine located at:

http://tos.org/oceanography/permissions.html

Best regards,
Vicky Cullen
Assistant Editor

On Oct 17, 2015, at 10:35 AM, Marco Brunoldi wrote:

> Goodmorning,
> with this email I request permission for the open-access journal PLOS ONE to publish **Fig.1 (a), pag.39**, of the paper corresponding to the **reference reported below**, under the Creative Commons Attribution License (CCAL) CC BY 3.0 (http://creativecommons.org/licenses/by/3.0/us/). Please be aware that this license allows unrestricted use and distribution, even commercially, by third parties. Please reply and provide explicit written permission to publish **Fig.1 (a), pag.39** under a CC BY license.
>
> **Reference**
> Mellinger DK, Stafford KM, Moore SE, Dziak RP, Matsumoto H. An overview of fixed passive acoustic observation methods for cetaceans. Oceanography. 2007;20(4):36-45. Available from: http://dx.doi.org/10.5670/oceanog.2007.03.
>
> Thanks in advance for your answer.
> My best regards
>
> Marco Brunoldi
>
> --
> _/_/_/_/_/_/_/_/_/_/_/_/_/_/_/_/
>
> Dr. Marco Brunoldi, PhD
> Dip. di Fisica
> Universita' di Genova
> Via Dodecaneso,33 I-16146
> Genova,Italy
> Tel. +39(0)103536270
> e-mail brunoldi@fisica.unige.it
>
> _/_/_/_/_/_/_/_/_/_/_/_/_/_/_/_/
