# Supplementary material for: A Permanent Automated Real-Time Passive Acoustic Monitoring System for Bottlenose Dolphin Conservation in the Mediterranean Sea
Source: PLoS One. 2016 Jan 20;11(1):e0145362. doi: 10.1371/journal.pone.0145362 (PMC4720475; doi:10.1371/journal.pone.0145362)
Supplement: S2 File — (PDF) [file pone.0145362.s002.pdf]

Genova, 17/10/2015

As the corresponding author for the submission of the manuscript titled *"A Permanent Automated Real-Time Passive Acoustic Monitoring System for Bottlenose Dolphin Conservation in the Mediterranean Sea"*, submitted to the open-access journal PLOS ONE, on the behalf of the manuscript's authors, I grant the permission for the open-access journal PLOS ONE to publish Figures 1, 2, 3, 4, 5, 6, 7, 9, 10, 11, 12, 13, 14, 15, 16, 17 under the Creative Commons Attribution License (CCAL) CC BY 3.0 (http://creativecommons.org/licenses/by/3.0/us/).

Note: none of the figures mentioned above is covered by any kind of copyright. The figures have been prepared by the authors for the manuscript.

Marco Brunoldi

[illegible]
